# Genome-wide association analysis of stripe rust resistance in modern Chinese wheat

**DOI:** 10.1186/s12870-020-02693-w

**Published:** 2020-10-27

**Authors:** Mengjie Jia, Lijun Yang, Wei Zhang, Garry Rosewarne, Junhui Li, Enian Yang, Ling Chen, Wenxue Wang, Yike Liu, Hanwen Tong, Weijie He, Yuqing Zhang, Zhanwang Zhu, Chunbao Gao

**Affiliations:** 1 Hubei Key Laboratory of Food Crop Germplasm and Genetic Improvement, Food Crops Institute, Hubei Academy of Agricultural Sciences/Hubei Engineering and Technology Research Center of Wheat/Wheat Disease Biology Research Station for Central China, Wuhan, 430064 China; 2 College of Life Sciences, Wuhan University, Wuhan, 430072 China; 3 Institute of Plant Protection and Soil Science, Hubei Academy of Agricultural Sciences, Wuhan, 430064 China; 4 Department of Plant Sciences, North Dakota State University, Fargo, North Dakota 58108-6050 USA; 5 Department of Jobs, Precincts and Regions, Agriculture Victoria, 110 Natimuk Road, Horsham, Victoria 3400 Australia; 6 International Maize and Wheat Improvement Center (CIMMYT), Apdo. Postal 6-641, 06600 Mexico D.F., Mexico; 7 Crop Research Institute, Sichuan Academy of Agricultural Sciences, Chengdu, 610066 China; 8 Hubei Collaborative Innovation Center for Grain Industry, Yangtze university, Jingzhou, 434025 China

**Keywords:** Marker-trait association, Single nucleotide polymorphism (SNP), *Triticum aestivum*, Yellow rust

## Abstract

**Background:**

Stripe rust (yellow rust) is a significant disease for bread wheat (*Triticum aestivum* L.) worldwide. A genome-wide association study was conducted on 240 Chinese wheat cultivars and elite lines genotyped with the wheat 90 K single nucleotide polymorphism (SNP) arrays to decipher the genetic architecture of stripe rust resistance in Chinese germplasm.

**Results:**

Stripe rust resistance was evaluated at the adult plant stage in Pixian and Xindu in Sichuan province in the 2015–2016 cropping season, and in Wuhan in Hubei province in the 2013–2014, 2016–2017 and 2018–2019 cropping seasons. Twelve stable loci for stripe rust resistance were identified by GWAS using TASSEL and GAPIT software. These loci were distributed on chromosomes 1B, 1D, 2A, 2B, 3A, 3B, 4B (3), 4D, 6D, and 7B and explained 3.6 to 10.3% of the phenotypic variation. Six of the loci corresponded with previously reported genes/QTLs, including *Sr2/Yr30/Lr27*, while the other six (*QYr.hbaas-1BS*, *QYr.hbaas-2BL*, *QYr.hbaas-3AL*, *QYr.hbaas-4BL.3*, *QYr.hbaas-4DL*, and *QYr.hbaas-6DS*) are probably novel. The results suggest high genetic diversity for stripe rust resistance in this population. The resistance alleles of *QYr.hbaas-2AS*, *QYr.hbaas-3BS*, *QYr.hbaas-4DL*, and *QYr.hbaas-7BL* were rare in the present panel, indicating their potential use in breeding for stripe rust resistance in China. Eleven penta-primer amplification refractory mutation system (PARMS) markers were developed from SNPs significantly associated with seven mapped QTLs. Twenty-seven genes were predicted for mapped QTLs. Six of them were considered as candidates for their high relative expression levels post-inoculation.

**Conclusion:**

The resistant germplasm, mapped QTLs, and PARMS markers developed in this study are resources for enhancing stripe rust resistance in wheat breeding.

**Supplementary information:**

**Supplementary information** accompanies this paper at 10.1186/s12870-020-02693-w.

## Background

Stripe rust (yellow rust), incited by obligate biotroph fungus *Puccinia striiformis* Westend. f. sp. *tritici* (*Pst*), is a significant disease of bread wheat (*Triticum aestivum* L.) worldwide. An outbreak will fast destroy green leaves and, in turn, dramatically reduce photosynthesis, resulting in stunted and weakened plants, reduced grain numbers per spike, shriveled grains, and lower grain weights. Grain yield losses of 100% can occur in fields sown to susceptible cultivars [1]. Annual grain yield losses caused by this disease are currently estimated to be 5.47 million tons [2].

The use of resistant cultivars is an environmentally friendly, economical, and effective way to manage this disease [3]. Thus, genetic studies on stripe rust resistance are of great significance. Host resistance is categorized into seedling or all-stage resistance and adult-plant resistance (APR) [4]. To date, 82 stripe rust resistance genes have been permanently designated [5], and many more have been given temporary names or quantitative trait locus (QTL) designations [6]. These genes were summarized in 2013 to more than 140 QTLs identified in various studies [7]. Out of the identified resistance genes, eight genes were isolated (*Yr36* [8], *Yr18* [9], *Yr10* [3], *Yr46* [10], *Yr5/YrSP*, *Yr7* [11], *Yr15* [12], and *YrAS2388R* [13]).

With the co-evolution of host and pathogen, epidemics of stripe rust follow a boom-and-bust cycle. Extensive use of single resistance genes usually leads to the emergence of new virulent *Pst* races, followed by subsequent disease epidemics. The breakdown of resistance genes *Yr1* in Bima 1 and *Yr9* in 1BL.1RS wheat cultivars caused huge economic losses in China’s wheat production [6]. Continuous gene discovery is urgently needed to enrich the resistance gene diversity to slow the boom-and-bust cycle. For the longer-term, quantitative resistances controlled by minor QTLs are encouraged to be pyramided to achieve broad-spectrum and durable resistance to stripe rust.

As a powerful tool for QTL mining, genome-wide association studies (GWAS) have been widely used for gene discovery of multiple traits of wheat, including stripe rust resistance in a worldwide collection of spring wheat landraces [14], Ethiopian wheat [15], International Maize and Wheat Improvement Center (CIMMYT) elite wheat [16], European winter wheat [17], northern Chinese wheat landraces [18], and Sichuan wheat [19].

In this study, we undertook a GWAS on stripe rust resistance in 240 wheat accessions to: 1) study the phenotypic variance of stripe rust response and 2) detect the genetic loci underlying the stripe rust resistance. The results should help to understand the genetic basis of stripe rust resistance in Chinese modern wheat cultivars and facilitate the improvement of stripe rust resistance through marker-assisted selection (MAS).

## Results

### Phenotypic variation

**Fig. 1:**
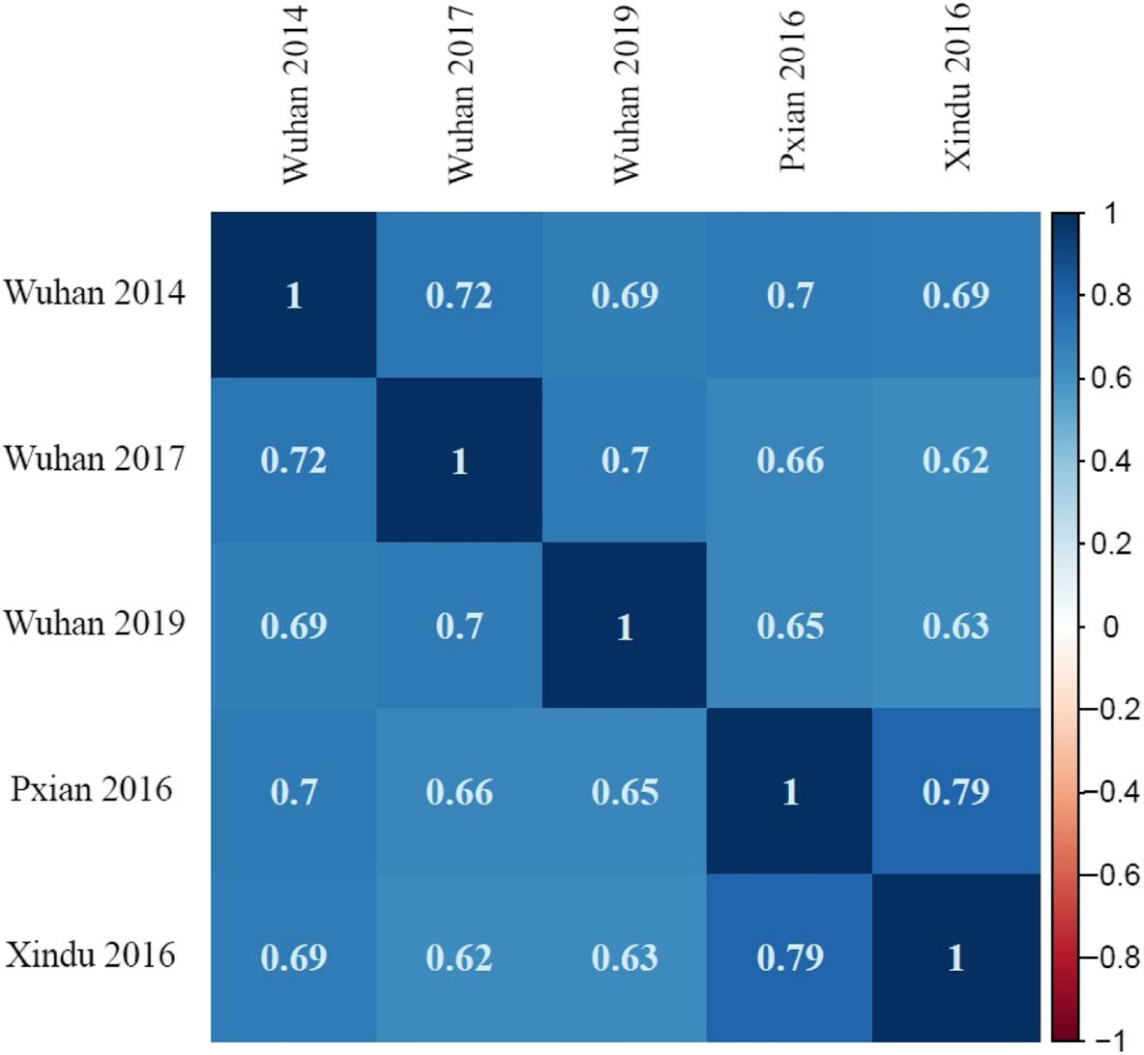
The pearson correlation coefficients of stripe rust severity in the natural populaiton among five environments

The best linear unbiased predictor (BLUP) values of stripe rust maximum disease severity (MDS) across five environments ranged from 8.8 to 89.1%, with an average of 49.8% (Additional file 1; Additional file 2), presenting a wide range of disease responses across the population. The levels of stripe rust symptoms varied across environments. The mean stripe rust MDS of the GWAS population were 43.6, 34.9, 71.2, 58.1 and 41.0% in Wuhan 2014, Wuhan 2017, Wuhan 2019, Xindu 2016, and Pixian 2016, respectively. The highest MDS was observed in Wuhan 2019 and disease severity in Wuhan 2017 was lighter than other environments. This might be attributed to not only the different weather conditions during inoculation and disease development but also the amount of pathogen in the natural environments. The variation of symptom levels might cause differences in the frequency distributions of the stripe rust MDS. Analysis of variance (ANOVA) revealed highly significant differences among genotypes, environments, and genotype × environment interactions (Table 1). Broad-sense heritability (*H*^*2*^) for MDS was estimated to be 0.91, indicating less environmental variance than genotypic variance; thus, this panel was suitable for further genetic analysis. High Pearson’s correlation coefficients (0.62–0.79) of stripe rust MDS were observed across five environments (Fig. 1). The highest correlation coefficient was observed between Pixian 2016 and Xindu 2016, and the lowest was between Wuhan 2017 and Xindu 2016.

**Table 1:** Analysis of variance of stripe rust severity of 240 wheat accessions

Source	*Df*	Sum squares	Mean squares	*P* value
Genotype	239	1,420,723.0	5944.4	< 0.0001
Environment	4	412,868.0	103,217.0	< 0.0001
Genotype×Environment	912	494,173.6	541.9	< 0.0001
Replication/Year	5	26,315.7	5263.1	< 0.0001

*H*^*2*^ = 0.91

**Fig. 2:**
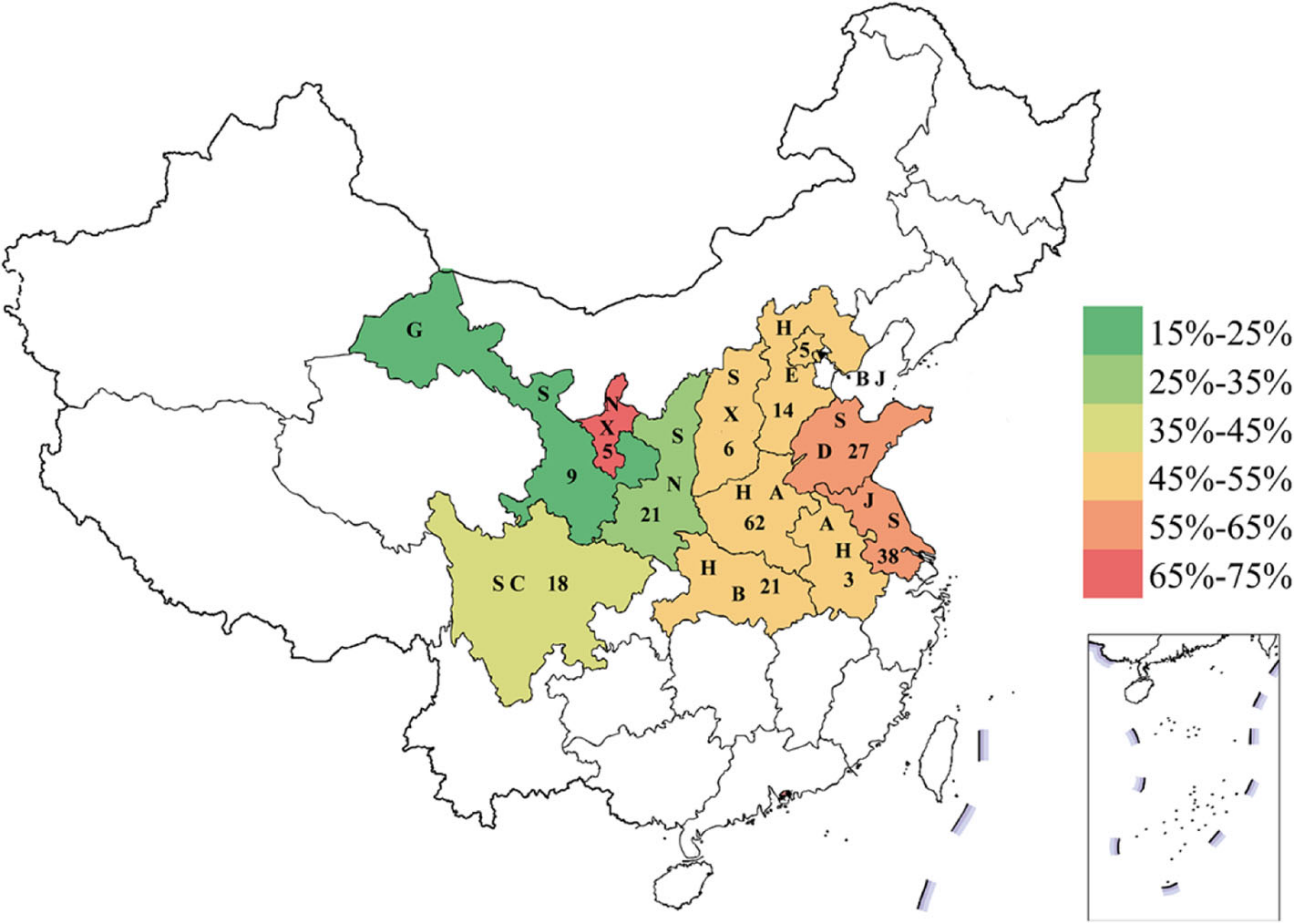
Sample numbers and averaged stripe rust severity of wheat accessions from different provinces of China. BJ, Beijing, 52.43%; HE, Hebei, 51.96%; SD, Shandong, 57.54%; SX, Shanxi, 51.04%; SN, Shaanxi, 35.38%; NX, Ningxia, 68.39%; GS, Gansu, 19.95%; JS, Jiangsu, 59.47%; AH, Anhui, 49.45%; HA, Henan, 55.06%; HB, Hubei, 49.86%; SC, Sichuan, 37.00%. Numeral in each province represents the number of cultivars (lines) sampled. Different colors represent corresponding stripe rust severity according to the legend. Map of China was obtained from http://bzdt.ch.mnr.gov.cn

Cultivars from Gansu had the highest stripe rust resistance with an average MDS of 20.0%, followed by Shaanxi and Sichuan at 35.4 and 37.0%, respectively. The cultivars from Henan (55.1%), Shandong (57.5%), and Jiangsu (59.5%) tended to be more susceptible (Fig. 2). CIMMYT lines presented good resistance to stripe rust with an average MDS of 28.6%.

### Marker coverage, genetic diversity, and population structure

After removing SNPs (single nucleotide polymorphism) with minor allele frequency (MAF) < 5% and missing rate > 20%, 14,578 SNPs were used for subsequent analyses. Of these, 5778 (39.6%), 6588 (45.2%), and 2212 (15.2%) were from the A, B and D genomes. The A (1.2 SNPs/Mb) and B (1.3 SNPs/Mb) genomes had higher marker density than the D (0.5 SNP/Mb) genome. Similarly, the A and B genomes had higher genetic diversity and polymorphism information content (PIC) values than the D genome (Additional file 3). A weak kinship was observed among cultivars in the association population [20]. The population was grouped into three sub-populations. The result was consistent with the geographic origin and pedigree. Most of the samples from Zone I (Northern Winter Wheat Zone), II (Yellow and Huai River Valleys Facultative Wheat Zone), VIII (Northwestern Spring Wheat Zone), and CIMMYT were grouped in sub-population I (Additional file 4), most of the Zone IV (Southwestern Autumn-Sown Spring Wheat Zone) cultivars in sub-population II, and the majority from Zone III in sub-population III. Some of the accessions from Zone II were also allocated to subgroups II and III indicating the germplasm exchange among different agro-ecological zones.

### Maker-trait associations (MTAs) and geographical distribution of favorable alleles

**Fig. 3:**
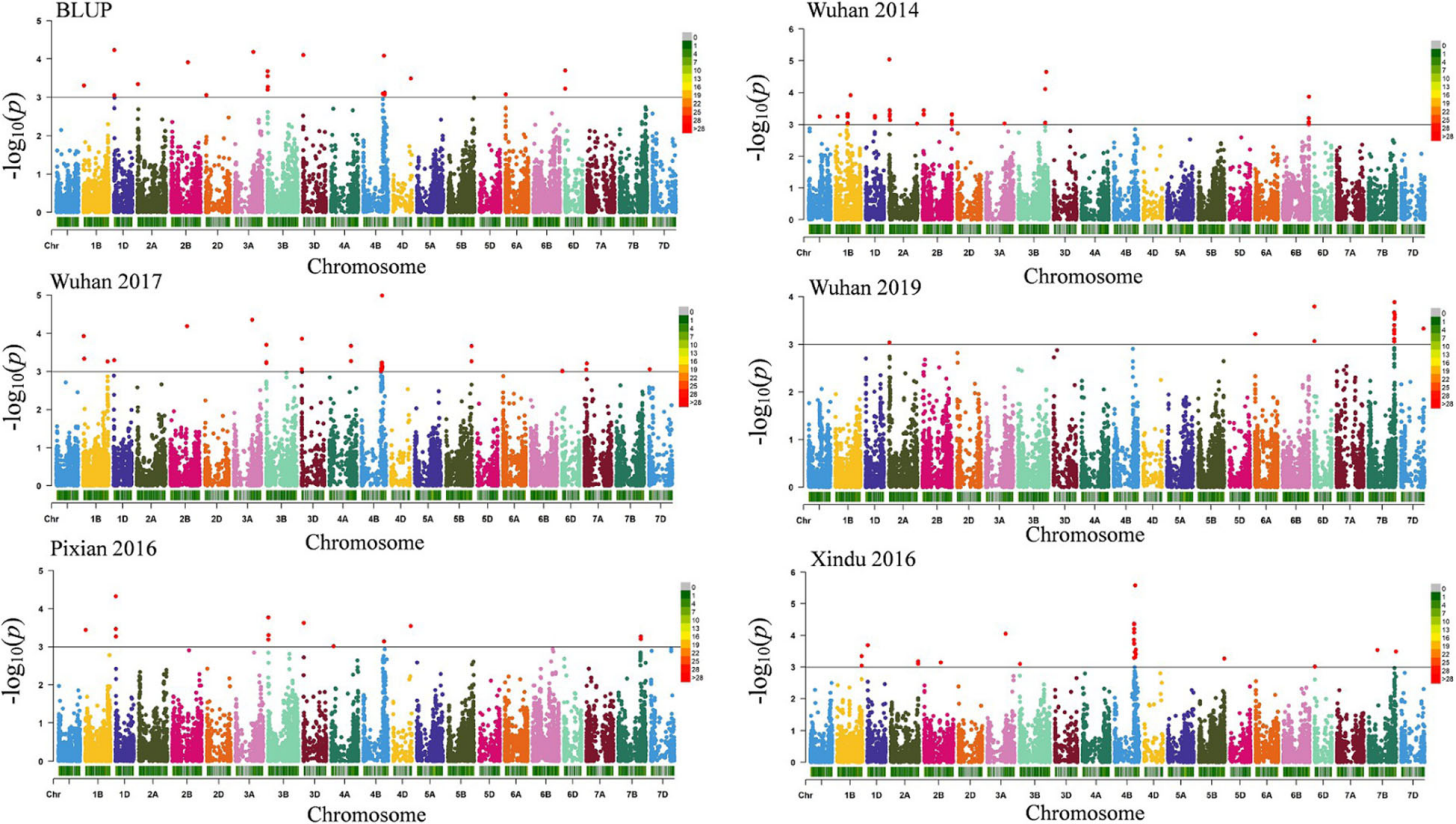
Manhattan plots for stripe rust severity of 240 wheat accessions performed by the mixed linear model with Tassel. The horizontal line indicates the threshold for significance

**Fig. 4:**
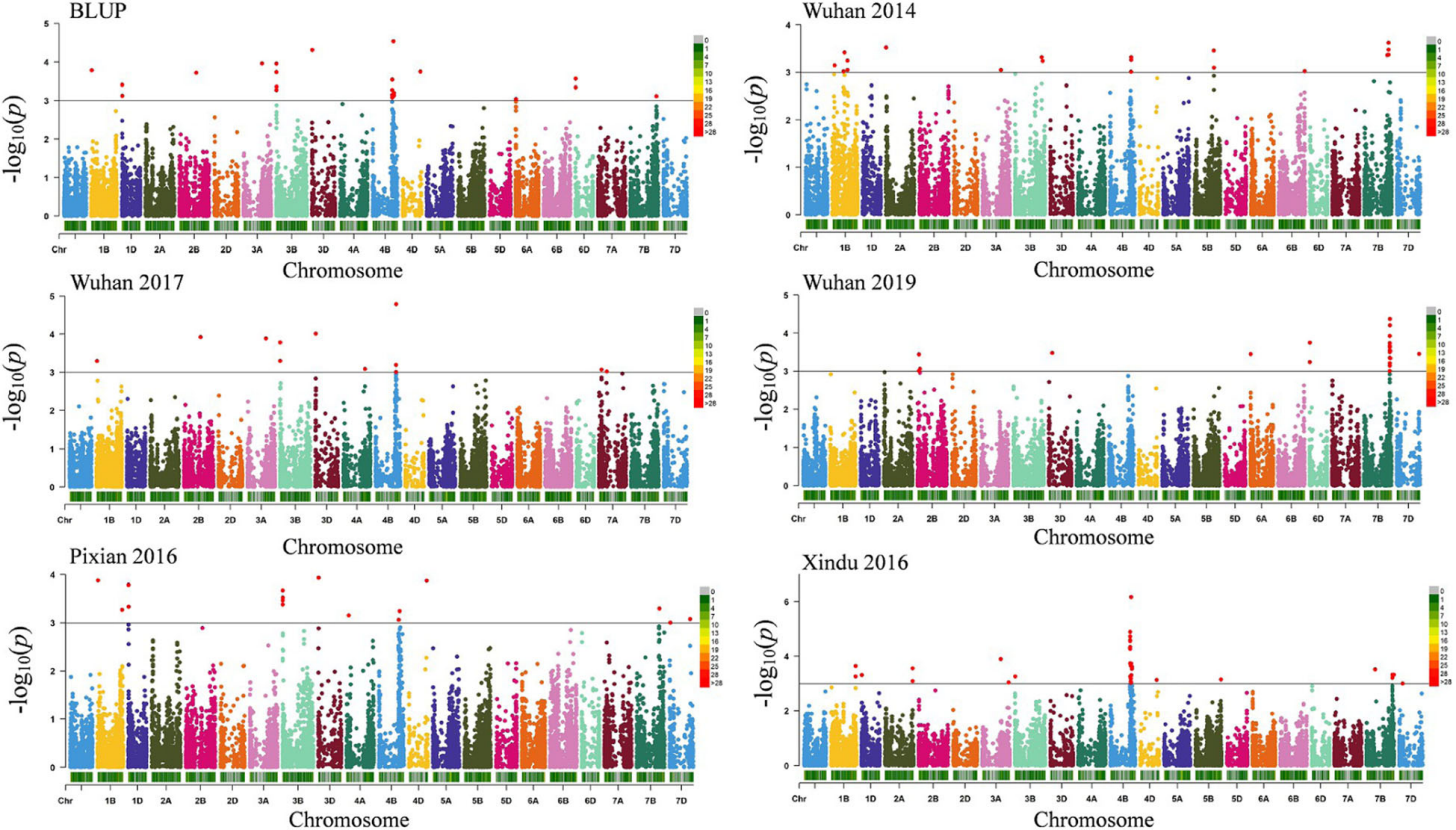
Manhattan plots for stripe rust severity of 240 wheat accessions performed by the mixed linear model with GAPIT. The horizontal line indicates the threshold for significance

Twelve stable loci for stripe rust resistance were identified (Table 2). These loci were distributed on chromosomes 1B, 1D, 2A, 2B, 3A, 3B, 4B (3), 4D, 6D, and 7B, explaining 3.6 to 10.3% of the phenotypic variation. Each locus was detected in two or more environments. Except for the stable loci, twenty-one QTLs were detected only in one environment on chromosomes 1B (3), 2A (2), 3B, 4A (2), 4B (4), 5B, 6B, 7B (6), and 7D (Additional file 5). TASSEL and Genomic Association and Prediction Integrated Tool (GAPIT) produced similar results (Fig. 3; Fig. 4). In contrast, more MTAs were detected by TASSEL than by GAPIT. The Q-Q plots obtained from the mixed linear model (MLM) in TASSEL v5.2.53 and GAPIT showed a well-controlled false-positive in this study, indicating the reliability of the MTAs (Additional file 6; Additional file 7).

**Table 2:** QTLs for stripe rust resistance detected by association study using TASSEL and GAPIT

QTL	Representative SNP	Position (Mb)	Allele ^a^	Environment	Method	*P*-value	*R*^*2* b^(%)
*QYr.hbaas-1BS*	*IWB45438*	9.7	G/A	BLUP	GAPIT	1.65E-04	4.7
				Pixian 2016	GAPIT	1.33E-04	5.5
				Wuhan 2017	GAPIT	5.09E-04	4.3
				BLUP	TASSEL	4.94E-04	5.7
				Pixian 2016	TASSEL	3.65E-04	6.2
				Wuhan 2017	TASSEL	1.17E-04	6.7
*QYr.hbaas-1DS*	*IWA1787*	8.6	C/T	BLUP	TASSEL	9.00E-04	4.9
				Pixian 2016	TASSEL	3.43E-04	6
*QYr.hbaas-2AS*	*IWB25308*	13.8	C/T	BLUP	TASSEL	4.58E-04	5.5
				Wuhan 2014	TASSEL	5.54E-04	6
				Wuhan 2019	TASSEL	9.11E-04	4.8
*QYr.hbaas-2BL*	*IWA586*	453.3	T/C	BLUP	GAPIT	1.91E-04	4.6
				Wuhan 2017	GAPIT	1.19E-04	5.3
				BLUP	TASSEL	1.23E-04	6.5
				Wuhan 2017	TASSEL	6.44E-05	6.9
				Xindu 2016	TASSEL	7.13E-04	5
*QYr.hbaas-3AL*	*IWB70396*	502.8	A/G	BLUP	GAPIT	1.10E-04	5
				Wuhan 2014	GAPIT	8.97E-04	4.4
				Wuhan 2017	GAPIT	1.29E-04	5.2
				Xindu 2016	GAPIT	1.27E-04	5.4
				BLUP	TASSEL	6.60E-05	7.4
				Wuhan 2014	TASSEL	9.30E-04	5.3
				Wuhan 2017	TASSEL	4.39E-05	7.4
				Xindu 2016	TASSEL	8.84E-05	7.1
*QYr.hbaas-3BS*	*IWB12253*	9.1	C/T	BLUP	GAPIT	4.40E-04	4.1
				Pixian 2016	GAPIT	2.98E-04	4.9
				Xindu 2016	GAPIT	5.50E-04	4.4
				BLUP	TASSEL	5.39E-04	5.2
				Pixian 2016	TASSEL	4.98E-04	5.5
				Xindu 2016	TASSEL	8.05E-04	4.9
*QYr.hbaas-4BL.1*	*IWB73717*	531.3	C/T	BLUP	GAPIT	8.36E-04	3.7
				Xindu 2016	GAPIT	2.79E-05	6.6
				Xindu 2016	TASSEL	8.16E-05	6.9
*QYr.hbaas-4BL.2*	*IWB63337*	558.1	T/C	Wuhan 2017	GAPIT	6.44E-04	4.1
				Xindu 2016	GAPIT	9.20E-04	4
				Wuhan 2017	TASSEL	5.80E-04	5.1
*QYr.hbaas-4BL.3*	*IWB32927*	579.4	T/C	BLUP	GAPIT	6.35E-04	3.9
				Wuhan 2014	GAPIT	4.80E-04	4.9
				Xindu 2016	GAPIT	2.91E-04	4.9
				BLUP	TASSEL	7.67E-04	5.2
				Xindu 2016	TASSEL	3.88E-04	5.8
*QYr.hbaas-4DL*	*IWB44356*	477.9	A/C	BLUP	GAPIT	1.77E-04	4.7
				Pixian 2016	GAPIT	1.35E-04	5.5
				BLUP	TASSEL	3.24E-04	5.9
				Pixian 2016	TASSEL	2.87E-04	6.2
*QYr.hbaas-6DS*	*IWB60233*	6.0	A/G	BLUP	GAPIT	4.57E-04	4.1
				Wuhan 2019	GAPIT	5.82E-04	4.2
				BLUP	TASSEL	5.97E-04	5.2
				Wuhan 2017	TASSEL	9.68E-04	4.7
				Wuhan 2019	TASSEL	8.57E-04	4.9
*QY.rhbaas-7BL*	*IWB64121*	704.3	T/C	BLUP	GAPIT	7.65E-04	3.8
				Wuhan 2019	GAPIT	2.24E-04	4.8
				Xindu 2016	GAPIT	6.17E-04	4.3
				Wuhan 2019	TASSEL	4.90E-04	5.5

^a^ Resistance alleles are underlined; ^b^ Phenotypic variation explained by the QTL

**Fig. 5:**
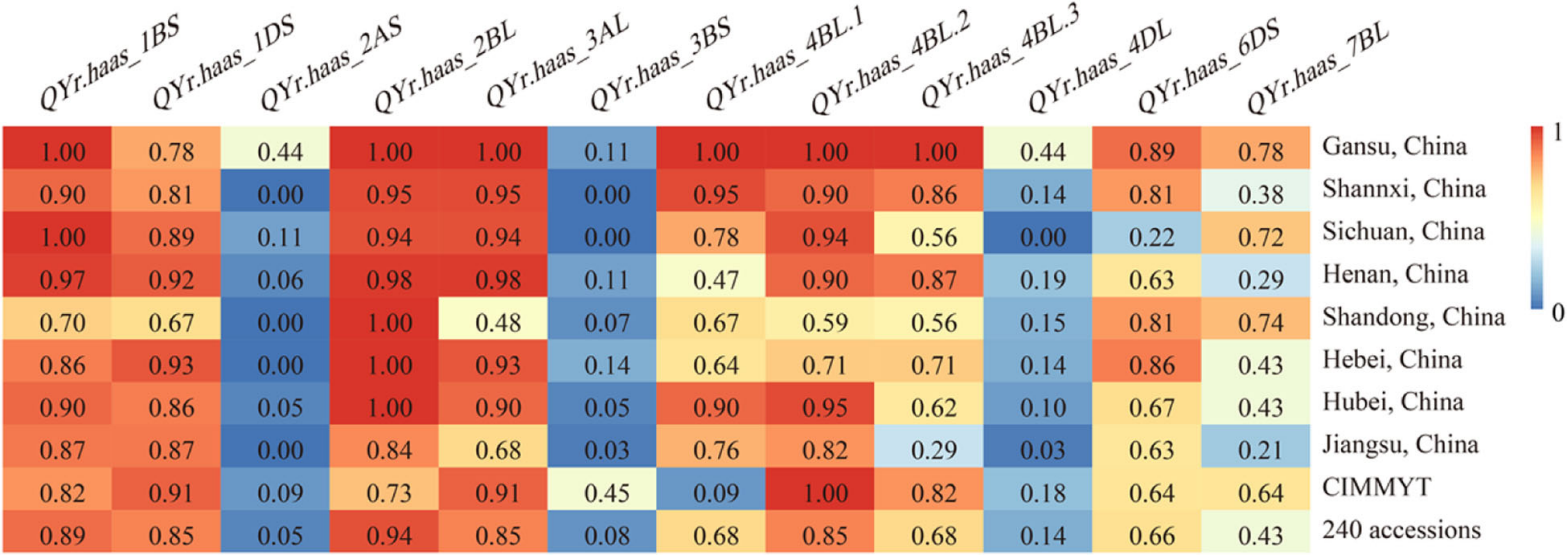
Allelic frequencies of the 12 stable QTLs in wheat cultivars from different provinces in China and CIMMYT lines. Provinces with number of wheat cultivars less than 9 were not shown

The favorable alleles of *QYr.hbaas-2AS*, *QYr.hbaas-3BS*, *QYr.hbaas-4DL*, and *QYr.hbaas-7BL* were rare in the present panel, with frequencies ranging from 0.05 to 0.43. The frequencies of the other seven QTLs were above 0.60. Relatively higher favorable allele frequencies (0.44) of *QYr.hbaas-2AS* and *QYr.hbaas-4DL* were observed in Gansu cultivars, compared with 0–0.19 in cultivars from other provinces. The favorable allele of *QYr.hbaas-3BS* was rare in Chinese wheat (0–0.14) compared with lines from CIMMYT (0.45). In contrast, *QYr.hbaas-4BL.1* was widely adapted in Chinese wheat but rare in CIMMYT lines (Fig. 5).

Fifty-one and 192 wheat accessions from the GWAS panel were speculated to contain *Yr62* and *Yr64*, respectively based on linked SSR markers analysis (Additional file 1). Six and 52 wheat accessions were tested to contain *YrSP* and *Yr7*, respectively using gene-specific markers. None of the wheat samples contains *Yr5* and *Yr15* indicating a lot of room for stripe rust resistance improvement in China.

### Relationship between stripe rust MDS and the number of favorable alleles

**Fig. 6:**
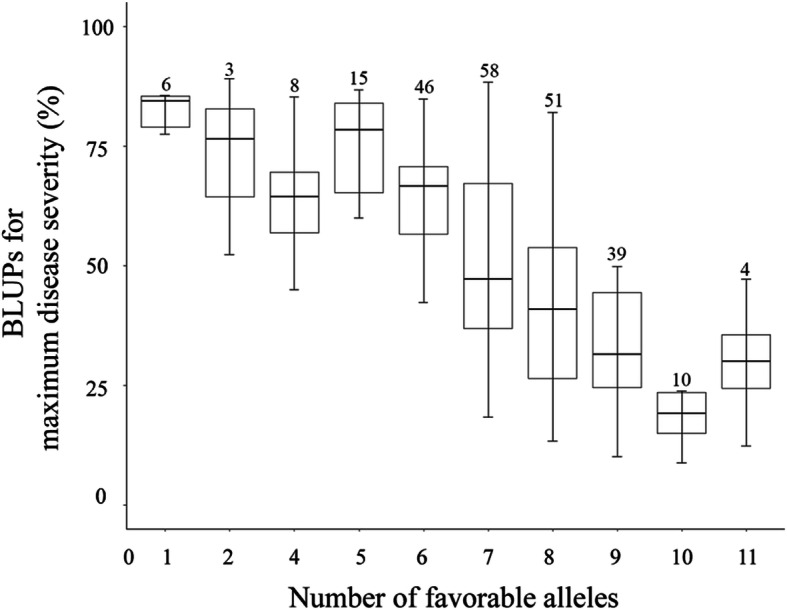
Averaged stripe rust maximum disease severity of lines with different number of favorable alleles of mapped QTLs. Arabic numerals on the bars indicates the number of accessions corresponding to each class

To illustrate the pyramiding effects of favorable alleles in different QTLs, we examined the number of favorable alleles of 12 mapped loci in each accession. The number of favorable alleles ranged from 1 to 12 (Additional file 1). Linear regression (*r*^*2*^ = 0.87) showed the dependence of disease severity on the number of favorable alleles (Fig. 6). Accessions with more favorable alleles, such as Lantian 15 (11 favorable alleles), Lantian 26 (11), Lantian 21 (10), Lantian 12 (10), and Zhongmai 12 (10) exhibited strong stripe rust resistance.

### PCR-based markers for mapped loci

A set of 11 penta-primer amplification refractory mutation system (PARMS) markers were successfully developed to detect the presence of stripe rust resistance QTLs (Additional file 8). Primers for these 11 markers are given in Additional file 9, and the protocols are described in Additional file 10. Developed markers were validated using the 240 GWAS accessions; the results produced very low frequencies of inconsistency (3.7–5.9%) with chip data.

### Candidate genes predicted

With TPMs (transcripts per kilobase million) above 0.5, three genes encoding NBS-LRR (nucleotide-binding site-leucine-rich repeat) proteins and two genes encoding receptor-like kinase (RLK) were identified in the region of *QYr.hbaas-1BS* (Additional file 11). Genes encoding disease resistance protein RPM1 and NBS-LRR might be candidates for *QYr.hbaas-1DS.* In the *QYr.hbaas-2AS* region, we identified four NBS-LRR genes. A TIR-NBS-LRR gene was found near *QYr.hbaas-2BL.* Kinase genes might be candidates for *QYr.hbaas-3AL*, *QYr.hbaas-4BL.3*, and *QYr.hbaas-4DL.* Two RLK genes were also identified at *QYr.hbaas-4DL.* Four genes encoding glycosyltransferase and an ATP-binding cassette gene were identified at *QYr.hbaas-3BS.* For *QYr.hbaas-6BS*, we identified three RLK genes and one gene encoding stress response NST1-like protein. The highest relative expression levels of these candidate genes ranged from 0.7 to 6.0. Compared to the expression at 0 hpi, the highest relative expression levels of *TraesCS1B01G020600*, *TraesCS1B01G020900* (*QYr.hbaas-1BS*), *TraesCS3B01G021100*, *TraesCS3B01G022000 (QYr.hbaas-3BS)*, TraesCS6D01G013600, *TraesCS6D01G014300* (*QYr.hbaas-6DS*) exceeded 1.5 which indicates their strong candidacy.

## Discussion

### Phenotypic variation and genetic diversity of stripe rust resistance

The levels of resistance to stripe rust in different provinces could be assessed with the mean stripe rust MDS. Samples from Gansu, Shaanxi, and Sichuan had the highest levels of resistance to stripe rust in China. This result is in accordance with the severe occurrence of stripe rust and substantial resistance-breeding efforts in these three provinces.

Additionally, CIMMYT germplasm showed high level of resistance to stripe rust. This is partly due to the long history of breeding for durable resistance to stripe rust at CIMMYT. The pathologists and wheat breeders from CIMMYT have focused on partial resistance for more than 30 years. This resistance source has been successfully used in the spring wheat region in China, such as Sichuan province [19]. The different resistance backgrounds of Chinese wheat in the CIMMYT germplasm suggest its usefulness in wheat breeding in China.

According to the favorable alleles frequency analysis, resistant stocks from Gansu, Shaanxi, Sichuan and CIMMYT carry different resistance loci. The high resistance level and diversity of resistance genes make them valuable sources of stripe rust resistance in breeding. This diversity also allows wheat breeders to pyramid various QTLs to achieve high level, broad-spectrum, and even durable resistance to stripe rust.

### Novelty of the mapped QTLs

*QYr.hbaas-1BS* was mapped at the distal region of 1BS (9.7 Mb). This chromosome arm is rich with stripe rust resistance genes/QTLs, including *Yr64* [21] and *Yr15* [12]. Most of the previously mapped loci are far from *QYr.hbaas-1BS* based on physical position (Additional file 12). The genotype of linked marker for *Yr64* is inconsistent with *QYr.hbaas-1BS* suggesting they are not the same loci. *QYr.hbaas-1BS* is different from *Yr15* either as no sample in the GWAS panel was detected to contain this gene based on gene-specific markers. Zegeye et al. [22] detected a QTL on 1BS (9.1 Mb) for stripe rust resistance by GWAS in synthetic hexaploid wheat, which is close to *QYr.hbaas-1BS*. However, this resistance source is unlikely to be widely adopted in Chinese wheat, and might differ from *QYr.hbaas-1BS* as the frequency of *QYr.hbaas-1BS* is very high in this wheat panel. Thus, *QYr.hbaas-1BS* is considered a new locus for stripe rust resistance.

*QYr.hbaas-1DS* overlapped *QYr.nwafu-1DS.1*, *QYrst.orr-1DS*, and *QYr.sun-1D* [23–25] with distances of 6.1, 8.2, and 8.3 Mb, respectively. Further studies are needed to clarify the relationships among the these QTLs on 1DS (Additional file 12).

Numerous genes/QTLs for stripe rust resistance on chromosome 2AS (Additional file 12) have been reported. Most of them are located in a 3.8–19.1 Mb interval, including *Traes_2AS_6BC67DD45*, *Traes_2AS_A477CDA77*, *Traes_2AS_6CE6AB560* [16], *Yr2A.1PBL*, *Yr2A.2PBL*, *Yr2A.3PBL*, *Yr2A.4PBL* [26], *QYr.tam-2A* [27], *QYr.tsw-2A.3* [28], *YrCH86* [29], *YrZM175* [30], *QYr.sun-2A* [31], *QYr.ufs-2A* [32], *YrR61* [33] and a QTL linked to *IWB57199* [15]. *QYr.hbaas-2AS* mapped in the present study is also located in this region.

On 2BL, *Yr5*/*YrSP*, *Yr7* [34, 35], and many other loci were mapped or isolated (Additional file 12). Among them, a QTL associated with *wsnp_JD_c744_1111659* is the closest to *QYr.hbaas-2BL*, with a distance of 126.7 Mb. The distinct locations between *QYr.hbaas-2BL* and other reported loci on 2BL indicated that *QYr.hbaas-2BL* is a new QTL for stripe rust resistance. The genotypes of gene-specific markers for *Yr5*, *YrSP*, and *Yr7* were distinct from *QYr.hbaas-2BL* further indicating their difference.

*QYr.dms-3A* [36], *QYrPI182103.wgp-3AL* [37], *QYrdr.wgp-3AL* [38], *QYr.nwafu-3AL* [23], QTLs linked to *wsnp_JD_c14691_14352459* [39], *IWB11855* [40], and *wsnp_RFL_Contig4814_5829093* [22] were mapped on 3AL (Additional file 12). Of these, the QTL linked to *wsnp_JD_c14691_14352459* is the closest to *QYr.hbaas-3AL,* with a distance of 30.2 Mb. This distance indicates that *QYr.hbaas-3AL* might be a new QTL for stripe rust resistance.

*QYr.hbaas-3BS* is close to a well-characterized pleiotropic locus *Sr2*/*Yr30*/*Lr27* [41] with a distance of 3 Mb. *Sr2* has been extensively used at CIMMYT to achieve durable resistance to multiple diseases. The highest frequency of *QYr.hbaas-3BS* in the CIMMYT accessions also indicates that this QTL might be *Sr2*/*Yr30*/*Lr27*.

On wheat chromosome 4BL, *QYrhm.nwafu-4B* [42] and *Yr62* [43] overlapped *QYr.hbaas-4BL.1* and *QYr.hbaas-4BL.2*, whereas no reported QTL was associated with *QYr.hbaas-4BL.3* based on physical position. Thus, *QYr.hbaas-4BL.3* is considered a new locus for stripe rust resistance. However, the genotypes of the representative SNPs for *QYr.hbaas-4BL.1* and *QYr.hbaas-4BL.2* were not consistent with the linked marker *Xgwm251* for *Yr62* with very low *R*^*2*^*.*

On 4DL, a QTL linked to *Xwmc399* (484.7 Mb) in oligoculm wheat [44], was close to *QYr.hbaas-4DL*. Most of the varieties used in China come from CIMMYT, while the oligoculm variety is from Israel; it is unlikely that oligoculm is used in China, so this QTL may not be the QTL associated in this study. Although the two QTLs were mapped to similar regions, they appear to have different origins. Thus, *QYr.hbaas-4DL* is probably new.

The QTL linked with *IWA167* [45], *QYr.sicau-6D* [46], and *QYr.ufs-6D* [32] were mapped on chromosome 6DS. None was close to *QYr.hbaas-6DS*, indicating that *QYr.hbaas-6DS* is probably a new locus.

Several stripe rust QTLs were mapped near *QYr.hbaas-7BL*, including *QYr.nwafu-7BL* [47], *Qyr.saas-7B* [48], *Qyrsicau-7BL* [19], *QYr.nwafu-7BL* [23], *QYr.caas-7BL.2* [49], and a QTL flanked by *wPt-4342* and *wPt-8921* [50].

### Application of resistant germplasm and MTAs in breeding for stripe rust resistance

The GWAS population represents a large proportion of the diversity of recently cultivated wheat in China. Fifty-four cultivars from the population have achieved a peak annual acreage of 1 × 10^5^ ha in the period 2000–2016 (http://202.127.42.47:6006/Home/BigDataIndex). Nine of them show good resistance to stripe rust with BLUE values of MDS under 30%. Interestingly, five of the nine resistant cultivars, Zhoumai 18, Zhoumai 22, Aikang 58, Jimai 22, and Liangxing 99, have also been used as founder parents in corresponding regions in recent years. This indicates that resistance is vital for parent selection in stripe rust-prone areas.

Average stripe rust MDS decreases dramatically when QTL number increases according to the regression of QTL number and the phenotype with a slop of − 5.4. This result indicates a promising effect of detected QTLs for stripe rust resistance. However, validations are needed in future studies. To further validate the effect of the mapped QTLs, the linked markers can be tested in different natural and bi-parental populations. Resistant accessions with potentially new QTLs could be used to produce bi-parental populations for further studies.

Grain yield and end-use quality are the main objectives for most breeding programs, however, resistance or tolerance to abiotic and biotic stresses is also a major concern for grain yield stabilization. As a minor QTL alone does not provide adequate resistance, pyramiding minor loci is necessary to achieve an acceptable level of resistance. For the long term, resistance QTLs are worth being gradually pyramided into founder parents for further improvement in stripe rust prevalent regions. PARMS markers developed in the present study will expedite this procedure with advantages of high throughput, low cost, and stability. Furthermore, the DNA template for PARMS can be prepared with alkaline lysis, which is convenient and time-saving [51]. In the stripe rust prevalent area, minor QTLs can also be pyramided through phenotypic selection which was verified to be feasible at CIMMYT [52]. Further, well-characterized resistance genes *Yr5* and *Yr15* effective against prevalent Chinese *Pst* races have not been incorporated in Chinese wheat cultivars. Combining these genes with minor QTLs will be another solution for stripe rust resistance breeding.

When selecting the resistance alleles of stripe rust QTLs through MAS, a large part of the respective chromosome will be fixed as identical by descent (IBD) which means the surrounding genes from the donor will be introduced along with the target QTLs. From this point of view, the resistant donors should be carefully selected not only for the target trait but also the agronomic performance to minimize linkage drag. Compared with introduced germplasm, using widely planted cultivars in the corresponding region may lower the risks of linkage drag. To avoid narrowing down the genetic diversity of stripe rust resistance, introducing resistant cultivars from other agro-ecological regions can enrich the resistance gene pool. When using these unadapted donors, 1–2 backcross with large populations will help getting rid of undesired traits.

Breeding for quantitative resistance is a challenge due to its complex inheritance. Genomic selection can facilitate parental selection, potentially accurately predict the phenotypes, and increase the genetic gain. Mean prediction accuracies reached 0.34 to 0.71 for stripe rust APR using different genomic prediction models [53] indicating its effectiveness in wheat breeding for stripe rust resistance. Markers for validated QTLs and well-characterized genes can used as fixed effects in genomic selection models to increases the prediction accuracy.

## Conclusions

In the present study, we identified 12 stable stripe rust resistance loci using the wheat 90 K SNP assay. Six stripe rust resistance loci *QYr.hbaas-1BS*, *QYr.hbaas-2BL*, *QYr.hbaas-3AL*, *QYr.hbaas-4BL.3*, *QYr.hbaas-4DL*, and *QYr.hbaas-6DS* are probably novel. Eleven PARMS markers associated with seven mapped QTLs were developed. The resistant germplasm, mapped QTLs, and developed PARMS markers could be employed in stripe rust resistance breeding.

## Methods

### Plant materials

The wheat association panel comprises 240 geographically diverse cultivars and elite lines (Additional file 1; Fig. 2), of which, 229 were from 12 provinces from China: Henan (62), Jiangsu (38), Shandong (27), Hubei (21), Shannxi (21), Sichuan (18), Hebei (14), Gansu (9), Shanxi (6), Beijing (5), Ningxia (5), and Anhui (3). This collection represents the currently cultivated wheat cultivars in China with more than 50 cultivars planted on at least 100,000 ha per year, including predominant cultivars Aikang 58, Jimai 22, Xinong 979, and Zhoumai 22. These Chinese wheat accessions were collected by Lijun Yang and Zhanwang Zhu from corresponding institutions with their permissions. The other 11 are ten elite lines from CIMMYT and an Australian cultivar, which were provided by David Bonnett under permission from CIMMYT.

### Disease assessment

All 240 accessions were evaluated for their response to stripe rust at the adult plant stage in Pixian (30°05′N, 102°54′E) and Xindu (30°83′N, 104°15′E) in Sichuan province, China, during the 2015–2016 cropping season (hereafter referred to as Pixian 2016 and Xindu 2016, respectively) and the Nanhu Experimental Station (30°48′N, 114°32′E) in Wuhan in Hubei province, China, during the 2013–2014, 2016–2017, 2018–2019 cropping seasons (noted as Wuhan 2014, Wuhan 2017, and Wuhan 2019, respectively). The field trials were conducted with randomized complete blocks with two replications. Each plot was sown in 1 m long paired rows. Stripe rust susceptible cultivar Mingxian 169 was planted around the nurseries as a disease spreader to facilitate uniform disease development. The disease spreader was inoculated with a mixture of prevalent *Pst* races CYR 32 and CYR 33 in the 2013–2014 cropping season in Wuhan and CYR 32 and CYR 34 in the other environments at the wheat shooting stage. CYR34 is the most virulent strain documented in China with an avirulence/virulence formula of *Yr5*, *Yr15* / *Yr1*, *Yr2*, *Yr3*, *Yr4*, *Yr6*, *Yr7*, *Yr8*, *Yr9*, *Yr10*, *Yr17*, *Yr18*, *Yr24/Yr26*, *Yr25*, *Yr27*, *Yr29*, *Yr30*, *Yr32*, *YrSP*, *YrA*, *YrSk*. CYR32 and CYR33 are avirulent to *Yr10* and *Yr24*/*Yr26* besides *Yr5* and *Yr15* compared with CYR34 [54]. Field studies were conducted in accordance with local practices.

The stripe rust response was scored as MDS when the severity of the susceptible cultivar Mingxian 169 reached the maximum level. The MDS under five environments and the BLUP across environments (hereafter referred to as BLUP) were used for subsequent analysis.

### Statistical analysis

The correlation coefficients were calculated using corrgram (https://github.com/kwstat/corrgram) package in R language (www.r-project.org). ANOVA was conducted with the ANOVA function in IciMapping v4.1 [55]. Broad-sense heritability (*H*^*2*^) for different traits was calculated using the following formula: *H*^2^ = ${\sigma}_G^2$ /(${\sigma}_G^2$ + ${\sigma}_{GE}^2$ /e + ${\sigma}_{\varepsilon}^2$ /(re)), where ${\sigma}_G^2$ is genotypic effect, ${\sigma}_{GE}^2$ is genotype by environment effect, ${\sigma}_{\varepsilon}^2$ is residual error, e is number of environments, and r is number of replications. BLU*P* values were calculated using ‘lme4’ package with R.

### Genotyping

The 240 wheat accessions were genotyped with an Illumina 90 K SNP array [56]. Calling and filtering of SNPs, kinship, and population structure analysis are described in our previous study [20]. Physical positions of SNPs were referred to the Chinese Spring reference genome sequences RefSeq v1.0 (http://www.wheatgenome.org). Genetic diversity [57], PIC, and MAF were computed by PowerMarker v3.25 [58].

Linked SSR markers *Xgwm251* [43] and *Xgdm33* [59] were used to test *Yr62* and *Yr64*, respectively in the GWAS panel. The PCR products were detected by 12% polyacrylamide gel electrophoresis. Gene-specific markers *Y15K1* [12] for *Yr15* and *Yr5-Insertion* for *Yr5* [11] were used to test corresponding genes. The PCR product was detected by 1.0% agarose gel electrophoresis. Linked KASP marker *Sr2_ger9 3p* was used to test *Yr30* [60]. Gene-specific KASP markers *Yr7-A*, *Yr7-D* for *Yr7* and *YrSP* for *YrSP* [11] were used to test corresponding genes.

### Genome-wide association analysis

GWAS was conducted using the MLM (PCA + K) in TASSEL v5.2.53 (http://www.maizegenetics.net/tassel) and GAPIT (http://zzlab.net/GAPIT). Due to the higher extent of linkage disequilibrium (LD) in wheat and the complex genetic architecture of stripe rust resistance, markers with an adjusted –log_10_ (*P*) ≥ 3.0 were regarded as significant for the trait. This threshold was also used in previous GWAS on stripe rust in bread wheat [17–19, 61]. Loci significant in at least two environments were reported in the results and considered stable QTLs. ‘CMplot’ package (https://github.com/YinLiLin/R-CMplot) was used to draw the Manhattan plots and quantile-quantile (Q-Q) plots with R. Alleles leading to lower MDS were referred to as favorable, whereas those leading to higher MDS were unfavorable. The frequencies of favorable alleles and their allelic effects were calculated based on the representative SNPs associated with the resistance loci.

### Development of PARMS markers for significant SNPs

To facilitate the use of mapped QTLs, significantly associated SNPs were developed as PARMS markers [51]. Primers were designed using PolyMarker (http://polymarker.tgac.ac.uk). Fluorescence signals were screened with PHERAstar^*Plus*^ (BMG LABTECH) and analyzed with KlusterCaller (LGC Genomics).

### Prediction of candidate genes

Annotated high confidence genes with a distance no more than 1 Mb from the representative SNPs of mapped QTLs were examined to consider the candidacy. The annotations of high confidence genes were referred to IWGSC RefSeq Annotation v1.0 (www.wheatgenome.org). Expressions of disease-related genes were analyzed in the public wheat expression database Triticeae Multi-omics Center (http://202.194.139.32) using the study performed by Zhang et al. (2014) [62]. Genes with a TPM > 0.5 were reported as the candidate genes for mapped QTLs. The highest expressions of these genes post-inoculation were also compared to those before inoculation to further confirm their candidacy.

## Supplementary information

**Additional file 1.** Names and origins of 240 wheat accessions.

**Additional file 2.** Frequency distributions for stripe rust maximum disease severity (MDS) of 240 wheat accessions in five environments.

**Additional file 3.** Marker density of the 240 wheat accessions genotyped with the 90 K SNP arrary.

**Additional file 4.** Principal components analysis (PCA) plots of 240 wheat accessions.

**Additional file 5.** Quantitative trait loci (QTLs) for stripe rust resistance significant only in one environment detected by genome-wide association study by TASSEL and GAPIT.

**Additional file 6.** Quantile-quantile plot for stripe rust maximum disease severity in 240 wheat accessions analyzed using the mixed linear model with Tassel.

**Additional file 7.** Quantile-quantile plot for stripe rust maximum disease severity in 240 wheat accessions analyzed using the mixed linear model with GAPIT.

**Additional file 8.** Profiles of 11 penta-primer amplification refractory mutation system markers for mapped stripe rust resistance loci.

**Additional file 9.** Primers for 11 penta-primer amplification refractory mutation system markers for seven stripe rust resistance loci.

**Additional file 10.** Protocols for 11 developed markers for seven stripe rust resistance loci.

**Additional file 11.** Candidate genes for stripe rust resistance QTLs.

**Additional file 12.** Reported QTLs/genes, linked markers and their physical positions used to align with QTLs mapped in the present study.

## Data Availability

The genotypic data generated from the 90 K wheat SNP array for the 240 wheat accessions is in the European Nucleotide Archive, PRJEB36125, https://www.ebi.ac.uk/ena/data/view/PRJEB36125. Other data supporting the results of this publication are included within the article and its additional files.

## Abbreviations

ANOVA: Analysis of variance
APR: Adult-plant resistance
BLUP: Best linear unbiased predictor
CIMMYT: International Maize and Wheat Improvement Center
GAPIT: Genomic Association and Prediction Integrated Tool
GWAS: Genome-wide association study
*H*^*2*^: Broad-sense heritability
IBD: Identical by descent
MAF: Minor allele frequency
MAS: Marker-assisted selection
MDS: Maximum disease severity
MLM: Mixed linear model
MTA: Maker-trait association
NBS-LRR: Nucleotide-binding site-leucine-rich repeat
PARMS: Penta-primer amplification refractory mutation system
PIC: Polymorphism information content
*Pst*: *Puccinia striiformis* f. sp. *Tritici*
QTL: Quantitative trait locus
RLK: Receptor-like kinase
SNP: Single nucleotide polymorphism
TPM: Transcripts per kilobase million
