# Supplementary material for: Genome-wide association analysis of stripe rust resistance in modern Chinese wheat
Source: BMC Plant Biol. 2020 Oct 27;20:491. doi: 10.1186/s12870-020-02693-w (PMC7590722; doi:10.1186/s12870-020-02693-w)
Supplement: Supplementary file 1 — Additional file 1. Names and origins of 240 wheat accessions. [file 12870_2020_2693_MOESM1_ESM.doc]

**Additional file 1** Names and origins of 240 wheat accessions

| No. | Cultivar (line) | Origin | Wheat zone ^a^ | Number of favorable alleles ^b^ | *Yr5* | *YrSP* | *Yr7* | *Yr15* | *Yr62* | *Yr64* | MDS ^c^ |
| --- | --- | --- | --- | --- | --- | --- | --- | --- | --- | --- | --- |
| GWAS1 | Emai 596 | Hubei, China | III | 9 | - | - | - | - | - | + | 31.4 |
| GWAS2 | Yang 05-117 | Jiangsu, China | III | 7 | - | - | - | - | + | + | 66.6 |
| GWAS3 | Yanzhan 4110 | Henan, China | II | 6 | - | - | - | - | - | + | 66.8 |
| GWAS4 | Chuanmai 42 | Sichuan, China | IV | 7 | - | - | + | - | - | + | 18.4 |
| GWAS5 | Shaannong 138 | Shannxi, China | II | 8 | - | - | - | - | - | + | 18.9 |
| GWAS6 | Emai 27 | Hubei, China | III | 7 | - | - | - | - | + | + | 59.9 |
| GWAS7 | Yang 06-144 | Jiangsu, China | III | 5 | - | - | - | - | + | + | 69.3 |
| GWAS8 | Lantian 13 | Gansu, China | VIII | 8 | - | - | - | - | - | + | 24.3 |
| GWAS9 | Lantian 17 | Gansu, China | VIII | 7 | - | - | - | - | - | + | 44.0 |
| GWAS10 | SYN1 | CIMMYT |  | 7 | - | - | + | - | - | + | 27.1 |
| GWAS11 | Luohan 6 | Henan, China | II | 7 | - | - | - | - | - | + | 78.8 |
| GWAS12 | Xiaoyan 22 | Shannxi, China | II | 8 | - | - | - | - | - | + | 80.9 |
| GWAS13 | Jingfumai 1 | Hubei, China | III | 7 | - | - | - | - | - | + | 45.1 |
| GWAS14 | Yangmai 12 | Jiangsu, China | III | 7 | - | - | - | - | - | + | 51.6 |
| GWAS15 | Ningchun 47 | Ningxia, China | VIII | 2 | - | - | - | - | - | + | 76.4 |
| GWAS16 | Neimai 8 | Sichuan, China | IV | 8 | - | - | + | - | - | + | 60.8 |
| GWAS17 | Xinmai 16 | Henan, China | II | 7 | - | - | + | - | - | + | 76.6 |
| GWAS18 | Mianyang 99-7 | Sichuan, China | IV | 5 | - | - | - | - | + | + | 86.7 |
| GWAS19 | Ping’an 6 | Henan, China | II | 8 | - | - | - | - | - | + | 40.8 |
| GWAS20 | Jinfeng 3 | Henan, China | II | 7 | - | - | - | - | - | - | 70.7 |
| GWAS21 | E’en 1 | Hubei, China | III | 9 | - | - | - | - | - | + | 79.9 |
| GWAS22 | Yumai 70-36 | Henan, China | II | 8 | - | - | - | - | + | + | 27.8 |
| GWAS23 | Mianmai 42 | Sichuan, China | IV | 6 | - | - | - | - | + | + | 66.5 |
| GWAS24 | Ningdong 11 | Ningxia, China | VIII | 6 | - | - | - | - | - | + | 68.8 |
| GWAS25 | Jimai 20 | Shandong, China | II | 9 | - | - | - | - | - | + | 30.7 |
| GWAS26 | Zhengmai 9694 | Henan, China | II | 9 | - | - | - | - | - | + | 49.9 |
| GWAS27 | Zhengyumai 958 | Henan, China | II | 6 | - | - | - | - | - | + | 73.3 |
| GWAS28 | Emai 580 | Hubei, China | III | 5 | - | - | + | - | - | + | 85.4 |
| GWAS29 | CJ9306 | Jiangsu, China | III | 6 | - | - | - | - | - | + | 64.7 |
| GWAS30 | Xiangmai 55 | Hubei, China | III | 9 | - | - | + | - | - | + | 40.8 |
| GWAS31 | Keda 9612 | Henan, China | II | 5 | - | - | - | - | - | + | 86.5 |
| GWAS32 | Yannong 22 | Shandong, China | II | 6 | - | - | - | - | - | + | 63.0 |
| GWAS33 | Qingfeng 1 | Shandong, China | II | 4 | - | - | - | - | - | + | 74.7 |
| GWAS34 | Xumai 27 | Jiangsu, China | II | 7 | - | - | - | - | - | + | 69.5 |
| GWAS35 | Zhoumai 17 | Henan, China | II | 9 | - | - | - | - | - | - | 15.6 |
| GWAS36 | E’en 6 | Hubei, China | III | 6 | - | - | + | - | + | + | 70.2 |
| GWAS37 | Zhoumai 23 | Henan, China | II | 9 | - | - | - | - | - | - | 76.1 |
| GWAS38 | Mianmai 1403 | Sichuan, China | IV | 8 | - | - | - | - | - | + | 15.2 |
| GWAS39 | Xikemai 4 | Sichuan, China | IV | 7 | - | - | - | - | - | + | 21.9 |
| GWAS40 | XK0106-1-0806 | Sichuan, China | IV | 8 | - | - | + | - | - | + | 22.3 |
| GWAS41 | Yangmai 22 | Jiangsu, China | III | 6 | - | - | - | - | - | + | 62.2 |
| GWAS42 | Zhongmai 1 | Henan, China | II | 6 | - | - | + | - | - | + | 77.4 |
| GWAS43 | Kenong 9204 | Hebei, China | II | 9 | - | - | - | - | - | + | 27.2 |
| GWAS44 | Luomai 21 | Henan, China | II | 10 | - | - | - | - | - | - | 22.4 |
| GWAS45 | Yumai 49-168 | Henan, China | II | 7 | - | - | - | - | - | - | 73.6 |
| GWAS46 | Lantian 23 | Gansu, China | VIII | 9 | - | - | - | - | - | + | 15.6 |
| GWAS47 | Xiangmai 99 | Henan, China | II | 6 | - | - | - | - | - | + | 70.8 |
| GWAS48 | Wanmai 52 | Anhui, China | II | 8 | - | - | + | - | - | + | 49.5 |
| GWAS49 | Zhoumai 22 | Henan, China | II | 10 | - | - | - | - | - | - | 12.4 |
| GWAS50 | Ningdong 10 | Ningxia, China | VIII | 6 | - | - | - | - | - | + | 63.3 |
| GWAS51 | Zhoumai 16 | Henan, China | II | 8 | - | - | - | - | - | + | 43.7 |
| GWAS52 | Yunong 202 | Henan, China | II | 7 | - | - | + | - | - | + | 60.3 |
| GWAS53 | Shannong 664 | Shandong, China | II | 6 | - | - | + | - | - | + | 66.8 |
| GWAS54 | Shannong 189 | Shandong, China | II | 6 | - | - | + | - | - | - | 69.6 |
| GWAS55 | Yumai 52 | Henan, China | II | 7 | - | - | - | - | - | - | 70.9 |
| GWAS56 | Chuanmai 50 | Sichuan, China | IV | 6 | - | - | + | - | + | + | 45.3 |
| GWAS57 | Xinmai 18 | Henan, China | II | 7 | - | - | - | - | - | + | 23.0 |
| GWAS58 | Ning 7840 | Jiangsu, China | III | 8 | - | - | - | - | + | + | 32.1 |
| GWAS59 | Yangmai 158 | Jiangsu, China | III | 7 | - | - | + | - | + | + | 56.3 |
| GWAS60 | Yannong 24 | Shandong, China | II | 1 | - | - | - | - | - | + | 85.6 |
| GWAS61 | Chuanmai 52 | Sichuan, China | IV | 8 | - | - | + | - | - | - | 22.5 |
| GWAS62 | Xikemai 2 | Sichuan, China | IV | 7 | - | - | - | - | + | + | 25.1 |
| GWAS63 | 14FHBSN6404 | CIMMYT |  | 9 | - | - | + | - | - | + | 32.4 |
| GWAS64 | Taishan 21 | Shandong, China | II | 6 | - | - | - | - | + | + | 63.7 |
| GWAS65 | Xinong 979 | Shannxi, China | II | 6 | - | - | - | - | - | + | 45.7 |
| GWAS66 | Yang 07-49 | Jiangsu, China | III | 7 | - | - | - | - | - | + | 67.0 |
| GWAS67 | Linfen 138 | shanxi, China | II | 9 | - | - | - | - | - | + | 44.8 |
| GWAS68 | Han 3475 | Hebei, China | II | 9 | - | - | - | - | - | + | 31.3 |
| GWAS69 | Han 6172 | Hebei, China | II | 6 | - | - | + | - | - | + | 42.3 |
| GWAS70 | Jimai 30 | Hebei, China | II | 8 | - | - | + | - | - | - | 35.7 |
| GWAS71 | Liken 2 | Shannxi, China | II | 8 | - | - | - | - | - | + | 36.6 |
| GWAS72 | Xumai 29 | Jiangsu, China | II | 8 | - | - | - | - | - | + | 56.0 |
| GWAS73 | Wenmai 6 | Henan, China | II | 7 | - | - | + | - | + | + | 81.5 |
| GWAS74 | Xinmai 20 | Henan, China | II | 8 | - | - | + | - | - | - | 45.5 |
| GWAS75 | Yan 5158 | Shandong, China | II | 8 | - | - | - | - | + | + | 82.0 |
| GWAS76 | Lin Y867 | shanxi, China | II | 9 | - | - | - | - | - | + | 18.3 |
| GWAS77 | Emai 23 | Hubei, China | III | 8 | - | - | + | - | - | + | 65.9 |
| GWAS78 | Jining 16 | Shandong, China | II | 9 | - | - | - | - | - | + | 23.1 |
| GWAS79 | Shaan 715 | Shannxi, China | II | 9 | - | - | - | - | - | + | 28.3 |
| GWAS80 | Wanmai 38 | Anhui, China | II | 8 | - | - | + | - | - | - | 46.1 |
| GWAS81 | Wenmai 7 | Henan, China | II | 7 | - | - | - | - | - | - | 82.1 |
| GWAS82 | Yumai 48 | Henan, China | II | 8 | - | - | + | - | - | + | 52.0 |
| GWAS83 | Chuanmai 51 | Sichuan, China | IV | 7 | - | - | - | - | - | + | 35.0 |
| GWAS84 | Xinmai 22 | Henan, China | II | 8 | - | - | - | - | - | + | 13.4 |
| GWAS85 | Kenong 199 | Henan, China | II | 6 | - | + | - | - | + | + | 82.9 |
| GWAS86 | Lianmai 1 | Jiangsu, China | II | 4 | - | - | - | - | + | + | 50.8 |
| GWAS87 | Liangxing 99 | Hebei, China | II | 10 | - | - | - | - | - | + | 22.9 |
| GWAS88 | Shuangkang 7438 | Sichuan, China | IV | 9 | - | - | - | - | - | + | 26.9 |
| GWAS89 | Zhenmai 6 | Jiangsu, China | III | 6 | - | - | - | - | + | + | 72.4 |
| GWAS90 | 14FHBSN6418 | CIMMYT |  | 5 | - | - | - | - | - | + | 25.7 |
| GWAS91 | Yannong 19 | Shandong, China | II | 5 | - | - | - | - | - | + | 64.6 |
| GWAS92 | Chang 6359 | shanxi, China | II | 6 | - | - | - | - | - | - | 62.3 |
| GWAS93 | Xinong 3517 | Shannxi, China | II | 9 | - | - | - | - | - | + | 10.2 |
| GWAS94 | Lantian 18 | Gansu, China | VIII | 9 | - | - | - | - | - | + | 12.2 |
| GWAS95 | Ningmai 16 | Jiangsu, China | III | 6 | - | - | - | - | + | + | 68.1 |
| GWAS96 | Luohan 7 | Henan, China | II | 5 | - | - | - | - | + | + | 83.7 |
| GWAS97 | E’en 5 | Hubei, China | III | 6 | - | - | + | - | + | + | 65.5 |
| GWAS98 | Linfen 137 | shanxi, China | II | 9 | - | - | - | - | - | + | 34.7 |
| GWAS99 | 14FHBSN6405 | CIMMYT |  | 7 | - | - | - | - | - | + | 43.9 |
| GWAS100 | Wenmai 18 | Henan, China | II | 7 | - | - | - | - | - | + | 85.2 |
| GWAS101 | Wenmai 19 | Henan, China | II | 7 | - | - | - | - | - | - | 67.4 |
| GWAS102 | Zhenmai 9 | Jiangsu, China | III | 6 | - | - | - | - | + | + | 75.5 |
| GWAS103 | Jimai 38 | Hebei, China | II | 1 | - | - | - | - | + | + | 85.5 |
| GWAS104 | Lantian 15 | Gansu, China | VIII | 11 | - | - | - | - | - | + | 12.4 |
| GWAS105 | Emai 12 | Hubei, China | III | 7 | - | - | - | - | - | + | 41.4 |
| GWAS106 | Zhengmai 9023 | Henan, China | II | 8 | - | - | - | - | - | + | 49.0 |
| GWAS107 | Shijiazhuang 8 | Hebei, China | II | 6 | - | - | - | - | + | + | 71.4 |
| GWAS108 | Jingdong 17 | Beijng, China | I | 9 | - | - | - | - | - | + | 26.0 |
| GWAS109 | Xinong 9871 | Shannxi, China | II | 8 | - | - | - | - | - | + | 54.9 |
| GWAS110 | Jimai 21 | Shandong, China | II | 8 | - | - | - | - | - | + | 50.5 |
| GWAS111 | Yang 06G86 | Jiangsu, China | III | 6 | - | - | - | - | + | - | 70.3 |
| GWAS112 | Xinmai 11 | Henan, China | II | 7 | - | - | - | - | - | + | 40.5 |
| GWAS113 | Huaimai 20 | Jiangsu, China | II | 8 | - | - | - | - | - | + | 37.3 |
| GWAS114 | 14FHBSN6402 | CIMMYT |  | 8 | - | - | + | - | - | - | 15.5 |
| GWAS115 | Luomai 6010 | Henan, China | II | 9 | - | - | - | - | - | + | 48.0 |
| GWAS116 | Xinmai 9817 | Henan, China | II | 6 | - | - | - | - | - | + | 55.6 |
| GWAS117 | Xinmai 13 | Henan, China | II | 7 | - | + | - | - | - | - | 32.9 |
| GWAS118 | Yangmai 20 | Jiangsu, China | III | 7 | - | - | - | - | - | + | 58.9 |
| GWAS119 | Shaanmai 150 | Shannxi, China | II | 8 | - | - | - | - | - | + | 64.0 |
| GWAS120 | Zhoumai 18 | Henan, China | II | 10 | - | - | - | - | - | + | 23.8 |
| GWAS121 | Beijing 0045 | Beijng, China | I | 8 | - | - | - | - | - | - | 51.9 |
| GWAS122 | Yunong 035 | Henan, China | II | 9 | - | - | + | - | + | - | 47.5 |
| GWAS123 | Yangmai 15 | Jiangsu, China | III | 5 | - | - | - | - | - | - | 83.9 |
| GWAS124 | Hua 2533 | Hubei, China | III | 6 | - | - | + | - | - | + | 65.5 |
| GWAS125 | Ningmai 13 | Jiangsu, China | III | 2 | - | - | - | - | + | + | 89.1 |
| GWAS126 | Xiaoyan 6 | Shannxi, China | II | 1 | - | - | - | - | - | - | 25.3 |
| GWAS127 | Yang 07-129 | Jiangsu, China | III | 4 | - | - | - | - | - | + | 45.0 |
| GWAS128 | Zhengyumai 9987 | Henan, China | II | 9 | - | - | - | - | - | - | 43.2 |
| GWAS129 | Zhongmai 12 | Hebei, China | I | 10 | - | - | - | - | - | + | 15.3 |
| GWAS130 | Luoxin 998 | Henan, China | II | 9 | - | - | - | - | - | + | 27.7 |
| GWAS131 | Yang 07-44 | Jiangsu, China | III | 8 | - | - | - | - | - | - | 46.3 |
| GWAS132 | Fanmai 5 | Henan, China | II | 1 | - | - | - | - | - | + | 83.4 |
| GWAS133 | Sumai 3 | Jiangsu, China | III | 6 | - | - | - | - | - | + | 53.3 |
| GWAS134 | Hua 2566 | Hubei, China | III | 8 | - | - | - | - | - | - | 60.6 |
| GWAS135 | Gamenya | CIMMYT |  | 2 | - | - | - | - | - | + | 52.3 |
| GWAS136 | Luo 4-168 | Henan, China | II | 6 | - | - | + | - | - | + | 54.2 |
| GWAS137 | Ningmai 9 | Jiangsu, China | III | 4 | - | - | - | - | + | + | 85.3 |
| GWAS138 | Xikemai 5 | Sichuan, China | IV | 8 | - | - | - | - | + | + | 22.3 |
| GWAS139 | Xinmai 26 | Henan, China | II | 11 | - | - | - | - | + | + | 28.4 |
| GWAS140 | Xuke 1 | Henan, China | II | 8 | - | - | + | - | - | - | 77.6 |
| GWAS141 | Ningmai 8 | Jiangsu, China | III | 9 | - | - | - | - | - | + | 34.5 |
| GWAS142 | Zhengmai 004 | Henan, China | II | 11 | - | - | - | - | - | + | 47.2 |
| GWAS143 | Han 5316 | Hebei, China | II | 7 | - | - | - | - | - | - | 80.8 |
| GWAS144 | Zhongnong 2 | Beijng, China | I | 10 | - | - | - | - | - | + | 45.9 |
| GWAS145 | Weimai 8 | Shandong, China | II | 6 | - | - | - | - | - | - | 48.0 |
| GWAS146 | Zhenmai 168 | Jiangsu, China | III | 6 | - | - | - | - | + | + | 79.9 |
| GWAS147 | Shaanmai 139 | Shannxi, China | II | 9 | - | + | - | - | - | + | 33.1 |
| GWAS148 | 04 zhong 36 | Hubei, China | II | 7 | - | - | - | - | - | + | 48.2 |
| GWAS149 | Tainong 18 | Shandong, China | II | 5 | - | - | - | - | - | + | 84.0 |
| GWAS150 | Shannong 16 | Shandong, China | II | 6 | - | - | + | - | - | - | 43.1 |
| GWAS151 | Xinong 2000 | Shannxi, China | II | 9 | - | - | - | - | - | + | 47.0 |
| GWAS152 | Heng 115 | Hebei, China | II | 8 | - | - | - | - | - | + | 72.0 |
| GWAS153 | 14FHBSN6409 | CIMMYT |  | 9 | - | - | - | - | + | + | 18.5 |
| GWAS154 | Yumai 49-198 | Henan, China | II | 7 | - | - | - | - | - | - | 84.8 |
| GWAS155 | Yumai 69 | Henan, China | II | 7 | - | - | - | - | - | + | 39.7 |
| GWAS156 | Heng 4422 | Hebei, China | II | 7 | - | - | - | - | - | + | 65.8 |
| GWAS157 | Yangmai 13 | Jiangsu, China | III | 6 | - | - | - | - | + | + | 53.4 |
| GWAS158 | Xiangmai 25 | Hubei, China | III | 9 | - | - | + | - | - | + | 39.6 |
| GWAS159 | Ocoroni | CIMMYT |  | 8 | - | - | + | - | + | - | 27.2 |
| GWAS160 | Yang 07-15 | Jiangsu, China | III | 7 | - | - | - | - | - | + | 58.1 |
| GWAS161 | Zhengmai 98 | Henan, China | II | 6 | - | - | + | - | - | + | 84.9 |
| GWAS162 | Xinmai 9817 selection | Henan, China | II | 6 | - | - | - | - | - | + | 66.6 |
| GWAS163 | Aizao 791-99 selection | Henan, China | II | 6 | - | - | - | - | - | + | 69.0 |
| GWAS164 | Hemai 13 | Shandong, China | II | 6 | - | - | - | - | + | + | 59.7 |
| GWAS165 | Qinnong 142 | Shannxi, China | II | 8 | - | - | - | - | - | - | 23.2 |
| GWAS166 | 14FHBSN6411 | CIMMYT |  | 8 | - | - | + | - | - | + | 29.8 |
| GWAS167 | Emai 11 | Hubei, China | III | 7 | - | - | + | - | - | + | 46.3 |
| GWAS168 | Xiaoyan 166 | Shannxi, China | II | 8 | - | - | - | - | - | - | 25.8 |
| GWAS169 | Lianmai 2 | Jiangsu, China | II | 1 | - | - | - | - | - | + | 85.3 |
| GWAS170 | Yangmai 14 | Jiangsu, China | III | 6 | - | - | + | - | - | + | 73.1 |
| GWAS171 | Emai 352 | Hubei, China | III | 8 | - | - | - | - | - | + | 42.9 |
| GWAS172 | Zhengmai 366 | Henan, China | II | 8 | - | - | + | - | - | + | 33.1 |
| GWAS173 | Xinong 88 | Shannxi, China | II | 8 | - | - | - | - | - | + | 20.2 |
| GWAS174 | Jingzhou 66 | Hubei, China | III | 7 | - | - | + | - | + | + | 23.9 |
| GWAS175 | Emai 18 | Hubei, China | III | 7 | - | - | - | - | - | + | 36.8 |
| GWAS176 | Shaannong 78 | Shannxi, China | II | 6 | - | - | - | - | - | + | 30.6 |
| GWAS177 | Lin Y7287 | shanxi, China | II | 5 | - | - | - | - | + | + | 78.3 |
| GWAS178 | Lunxuan 987 | Beijng, China | I | 7 | - | - | - | - | - | + | 58.4 |
| GWAS179 | Jimai 22 | Shandong, China | II | 9 | - | - | - | - | - | + | 20.3 |
| GWAS180 | Shannong 15 | Shandong, China | II | 8 | - | - | + | - | + | + | 70.0 |
| GWAS181 | Yangfumai 2 | Jiangsu, China | III | 7 | - | - | - | - | - | + | 43.0 |
| GWAS182 | Shaannong 757 | Shannxi, China | II | 8 | - | - | - | - | - | + | 30.4 |
| GWAS183 | Yangmai 16 | Jiangsu, China | III | 9 | - | - | + | - | - | - | 44.1 |
| GWAS184 | Linyou 2069 | shanxi, China | II | 4 | - | - | - | - | - | + | 67.9 |
| GWAS185 | Yangmai 11 | Jiangsu, China | III | 7 | - | + | - | - | - | + | 49.5 |
| GWAS186 | Shaanmai 159 | Shannxi, China | II | 8 | - | - | - | - | - | + | 34.1 |
| GWAS187 | Jingdong 8 | Beijng, China | I | 5 | - | - | - | - | + | + | 80.0 |
| GWAS188 | Zhengnong 17 | Henan, China | II | 7 | - | - | - | - | - | - | 67.3 |
| GWAS189 | 14FHBSN6408 | CIMMYT |  | 9 | - | - | - | - | - | + | 19.4 |
| GWAS190 | Yumai 70 | Henan, China | II | 8 | - | - | - | - | - | + | 33.9 |
| GWAS191 | Yan 5286 | Shandong, China | II | 8 | - | - | - | - | + | + | 67.3 |
| GWAS192 | Hengguan 35 | Hebei, China | II | 7 | - | - | - | - | - | - | 60.4 |
| GWAS193 | Kaimai 18 | Henan, China | II | 9 | - | - | + | - | + | - | 48.7 |
| GWAS194 | Heng 136 | Hebei, China | II | 6 | - | - | - | - | + | + | 68.7 |
| GWAS195 | Yumai 10 | Henan, China | II | 5 | - | - | - | - | + | - | 74.0 |
| GWAS196 | Luohan 2 | Henan, China | II | 7 | - | - | - | - | - | + | 35.6 |
| GWAS197 | Ping’an 3 | Henan, China | II | 7 | - | - | + | - | - | + | 88.4 |
| GWAS198 | Jingmai 103 | Hubei, China | III | 7 | - | - | - | - | - | + | 33.2 |
| GWAS199 | Chuanmai 42 selection | Sichuan, China | IV | 7 | - | - | + | - | - | + | 37.3 |
| GWAS200 | Yannong 21 | Shandong, China | II | 8 | - | - | - | - | - | + | 66.6 |
| GWAS201 | Bainong 160 | Henan, China | II | 8 | - | - | + | - | - | - | 29.9 |
| GWAS202 | Shaan 627 | Shannxi, China | II | 9 | - | - | - | - | - | - | 26.8 |
| GWAS203 | Jinan 17 selection | Shandong, China | II | 7 | - | - | - | - | + | + | 65.0 |
| GWAS204 | E 07901 | Hubei, China | III | 10 | - | - | + | - | + | - | 23.8 |
| GWAS205 | Xiaoyan 107 | Shannxi, China | II | 8 | - | - | - | - | - | + | 18.1 |
| GWAS206 | Yumai 38 | Henan, China | II | 9 | - | - | - | - | - | + | 36.8 |
| GWAS207 | Yangmai 17 | Jiangsu, China | III | 4 | - | - | - | - | - | + | 58.9 |
| GWAS208 | Xinmai 19 | Henan, China | II | 7 | - | - | - | - | - | + | 34.1 |
| GWAS209 | Lumai 21 | Shandong, China | II | 5 | - | - | - | - | - | + | 60.0 |
| GWAS210 | Huaimai 17 | Jiangsu, China | II | 7 | - | - | - | - | - | + | 35.5 |
| GWAS211 | Mianmai 37 | Sichuan, China | IV | 7 | - | - | + | - | - | + | 41.7 |
| GWAS212 | Chuanmai 43 | Sichuan, China | IV | 7 | - | - | + | - | - | + | 41.2 |
| GWAS213 | Jinan 17 | Shandong, China | II | 9 | - | - | - | - | - | + | 47.2 |
| GWAS214 | Lantian 12 | Gansu, China | VIII | 10 | - | - | - | - | + | + | 14.9 |
| GWAS215 | Mianmai 185 | Sichuan, China | IV | 6 | - | - | + | - | - | + | 51.7 |
| GWAS216 | Lantian 21 | Gansu, China | VIII | 10 | - | - | - | - | + | + | 15.7 |
| GWAS217 | Jimai 19 | Shandong, China | II | 7 | - | - | - | - | + | + | 44.5 |
| GWAS218 | Lantian 26 | Gansu, China | VIII | 10 | - | - | - | - | - | + | 8.8 |
| GWAS219 | Shannong 8355 | Shandong, China | II | 6 | - | - | - | - | + | + | 43.5 |
| GWAS220 | Zhongyu 10 | Henan, China | II | 6 | - | - | - | - | - | - | 72.3 |
| GWAS221 | Yan 2415 | Shandong, China | II | 5 | - | - | - | - | - | + | 65.9 |
| GWAS222 | Shaan 253 | Shannxi, China | II | 6 | - | - | - | - | - | - | 69.0 |
| GWAS223 | Yuanfeng 175 | Shannxi, China | II | 9 | - | - | - | - | + | + | 20.2 |
| GWAS224 | Xinmai 208 | Henan, China | II | 6 | - | - | - | - | - | + | 76.2 |
| GWAS225 | Ningchun 4 | Ningxia, China | VIII | 4 | - | - | - | - | - | + | 66.1 |
| GWAS226 | Mianyang 99-3 | Sichuan, China | IV | 7 | - | - | - | - | - | + | 25.3 |
| GWAS227 | Lunong 116 | Shandong, China | II | 6 | - | - | - | - | + | - | 60.2 |
| GWAS228 | Wanmai 50 | Anhui, China | II | 8 | - | - | + | - | - | - | 52.8 |
| GWAS229 | Aikang 58 | Henan, China | II | 9 | - | + | - | - | - | - | 29.2 |
| GWAS230 | Ningchun 43 | Ningxia, China | VIII | 7 | - | - | + | - | + | - | 67.2 |
| GWAS231 | Wuhan 1 | Hubei, China | III | 7 | - | - | - | - | - | + | 40.8 |
| GWAS232 | Taishan 23 | Shandong, China | II | 5 | - | - | - | - | - | + | 34.0 |
| GWAS233 | Ningmai 11 | Jiangsu, China | III | 8 | - | - | - | - | - | + | 41.4 |
| GWAS234 | Lantian 22 | Gansu, China | VIII | 11 | - | + | - | - | - | + | 31.7 |
| GWAS235 | Mayoor | CIMMYT |  | 8 | - | - | + | - | - | + | 22.4 |
| GWAS236 | Xumai 216 | Jiangsu, China | II | 7 | - | - | - | - | - | - | 31.3 |
| GWAS237 | Zhongmai 9 | Hebei, China | I | 9 | - | - | - | - | - | + | 48.0 |
| GWAS238 | Pumai 10 | Henan, China | II | 7 | - | - | - | - | - | + | 39.2 |
| GWAS239 | Huaimai 18 | Jiangsu, China | II | 1 | - | - | - | - | - | + | 77.5 |
| GWAS240 | Yang 07-81 | Jiangsu, China | III | 4 | - | - | - | - | - | + | 62.7 |

^a^ I: Northern Winter Wheat Zone, II: Yellow and Huai River Valleys Facultative Wheat Zone, III: Middle and Lower Yangtze Valleys Autumn-Sown Spring Wheat Zone, IV: Southwestern Autumn-Sown Spring Wheat Zone, VIII: Northwestern Spring Wheat Zone; ^b^ Number of favorable alleles of mapped 12 stable QTLs detected with representative SNPs; ^c^ BLUPs of maximum disease severities (MDS) in field trials across five environments.
