# Supplementary material for: Genome-wide association analysis of stripe rust resistance in modern Chinese wheat
Source: BMC Plant Biol. 2020 Oct 27;20:491. doi: 10.1186/s12870-020-02693-w (PMC7590722; doi:10.1186/s12870-020-02693-w)

**Additional file 2** Frequency distributions for stripe rust maximum disease severity (MDS) of 240 wheat accessions in five environments


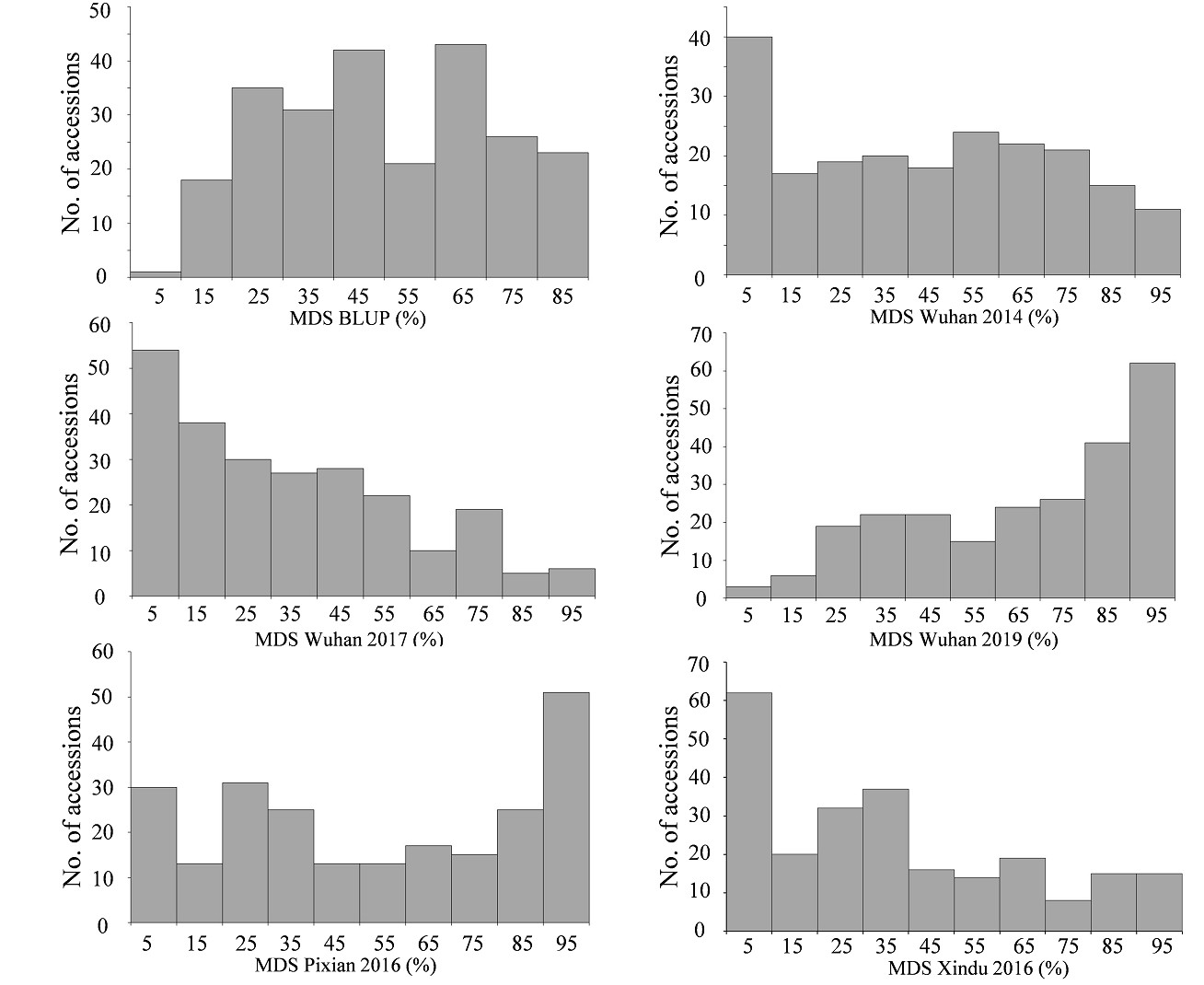

Supplement: Supplementary file 2 — Additional file 2. Frequency distributions for stripe rust maximum disease severity (MDS) of 240 wheat accessions in five environments. [file 12870_2020_2693_MOESM2_ESM.doc]
