# Supplementary material for: Genome-wide association analysis of stripe rust resistance in modern Chinese wheat
Source: BMC Plant Biol. 2020 Oct 27;20:491. doi: 10.1186/s12870-020-02693-w (PMC7590722; doi:10.1186/s12870-020-02693-w)
Supplement: Supplementary file 3 — Additional file 3. Marker density of the 240 wheat accessions genotyped with the 90 K SNP arrary. [file 12870_2020_2693_MOESM3_ESM.doc]

**Additional file 3** Marker density of the 240 wheat accessions genotyped with the 90K SNP arrary

| Chromosome | Length (Mb) | No. of SNPs | SNP density (SNPs/Mb) | Genetic diversity | PIC ^a^ |
| --- | --- | --- | --- | --- | --- |
| 1A | 594.10 | 996 | 1.68 | 0.334 | 0.271 |
| 1B | 689.85 | 1292 | 1.87 | 0.373 | 0.296 |
| 1D | 495.45 | 644 | 1.30 | 0.327 | 0.266 |
| 2A | 780.80 | 831 | 1.06 | 0.331 | 0.267 |
| 2B | 801.26 | 1050 | 1.31 | 0.329 | 0.265 |
| 2D | 651.85 | 359 | 0.55 | 0.315 | 0.258 |
| 3A | 750.84 | 770 | 1.03 | 0.347 | 0.279 |
| 3B | 830.83 | 878 | 1.06 | 0.357 | 0.286 |
| 3D | 615.55 | 257 | 0.42 | 0.307 | 0.254 |
| 4A | 744.59 | 584 | 0.78 | 0.323 | 0.262 |
| 4B | 673.62 | 483 | 0.72 | 0.326 | 0.265 |
| 4D | 509.86 | 106 | 0.21 | 0.311 | 0.253 |
| 5A | 709.77 | 905 | 1.28 | 0.364 | 0.291 |
| 5B | 713.15 | 1098 | 1.54 | 0.355 | 0.285 |
| 5D | 566.08 | 287 | 0.51 | 0.331 | 0.267 |
| 6A | 618.08 | 910 | 1.47 | 0.317 | 0.258 |
| 6B | 720.99 | 959 | 1.33 | 0.353 | 0.283 |
| 6D | 473.59 | 300 | 0.63 | 0.325 | 0.264 |
| 7A | 736.71 | 782 | 1.06 | 0.327 | 0.265 |
| 7B | 750.62 | 828 | 1.10 | 0.350 | 0.279 |
| 7D | 638.69 | 259 | 0.41 | 0.308 | 0.252 |
| A genome | 4934.89 | 5778 | 1.17 | 0.335 | 0.271 |
| B genome | 5180.31 | 6588 | 1.27 | 0.352 | 0.282 |
| D genome | 4432.06 | 2212 | 0.50 | 0.320 | 0.261 |
| All | 14547.26 | 14575 | 1.00 | 0.340 | 0.274 |

^a^ PIC, Polymorphism information content
