# Supplementary figures and images for: Genome-wide association analysis of stripe rust resistance in modern Chinese wheat
Source: BMC Plant Biol. 2020 Oct 27;20:491. doi: 10.1186/s12870-020-02693-w (PMC7590722; doi:10.1186/s12870-020-02693-w)

**Additional file 4** Principal components analysis (PCA) plots of 240 wheat accessions


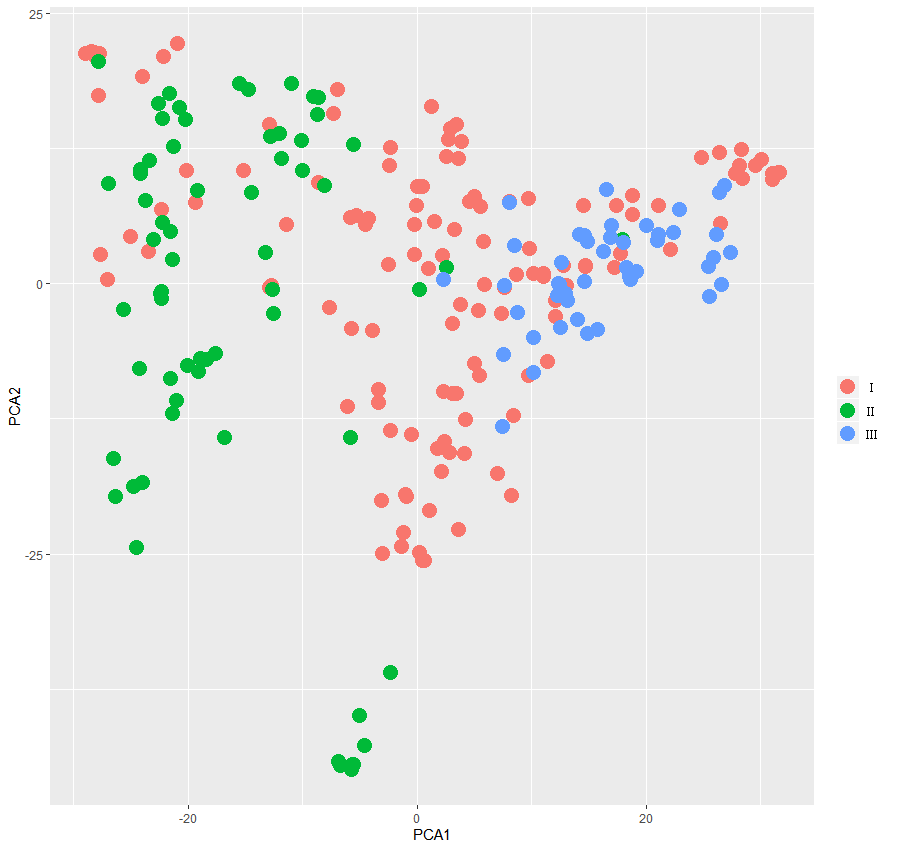

Supplement: Supplementary file 4 — Additional file 4. Principal components analysis (PCA) plots of 240 wheat accessions. [file 12870_2020_2693_MOESM4_ESM.doc]
