# Supplementary material for: Genome-wide association analysis of stripe rust resistance in modern Chinese wheat
Source: BMC Plant Biol. 2020 Oct 27;20:491. doi: 10.1186/s12870-020-02693-w (PMC7590722; doi:10.1186/s12870-020-02693-w)
Supplement: Supplementary file 5 — Additional file 5. Quantitative trait loci (QTLs) for stripe rust resistance significant only in one environment detected by genome-wide association study by TASSEL and GAPIT. [file 12870_2020_2693_MOESM5_ESM.doc]

**Additional file 5** Quantitative trait loci (QTLs) for stripe rust resistance significant only in one environment detected by genome-wide association study by TASSEL and GAPIT

| Chromosome | Representative SNP | Position (Mb) | Allele ^a^ | Environment | Method | *P* value | *R^2^* ^b^(%) |
| --- | --- | --- | --- | --- | --- | --- | --- |
| 1B | *IWB12562* | 57.1 | C/T | Wuhan 2014 | GAPIT | 7.19E-04 | 4.6 |
|  |  |  |  | Wuhan 2014 | TASSEL | 5.56E-04 | 5.7 |
|  | *IWB43992* | 330.2 | A/G | Wuhan 2014 | GAPIT | 3.83E-04 | 5.1 |
|  |  |  |  | Wuhan 2014 | TASSEL | 4.67E-04 | 5.8 |
|  | *IWB41428* | 666.9 | C/T | Xindu 2016 | GAPIT | 5.48E-04 | 4.4 |
|  |  |  |  | Xindu 2016 | TASSEL | 9.13E-04 | 4.8 |
| 2A | *IWA6922* | 4.8 | A/G | Wuhan 2014 | GAPIT | 3.00E-04 | 5.3 |
|  |  |  |  | Wuhan 2014 | TASSEL | 9.11E-06 | 10.5 |
|  | *IWB29388* | 758.4 | A/G | Xindu 2016 | GAPIT | 8.10E-04 | 4.1 |
|  |  |  |  | Xindu 2016 | TASSEL | 7.97E-04 | 5.1 |
| 3B | *IWA4146* | 730.3 | A/C | Wuhan 2014 | GAPIT | 4.83E-04 | 4.9 |
|  |  |  |  | Wuhan 2014 | TASSEL | 7.81E-05 | 7.5 |
| 4A | *IWA5457* | 65.5 | A/G | Pixian 2016 | GAPIT | 7.00E-04 | 4.3 |
|  |  |  |  | Pixian 2016 | TASSEL | 9.77E-04 | 5.1 |
|  | *IWB58408* | 597.9 | C/T | Wuhan 2017 | GAPIT | 8.16E-04 | 3.9 |
|  |  |  |  | Wuhan 2017 | TASSEL | 5.31E-04 | 5.2 |
| 4B | *IWB71823* | 532.6 | A/G | Xindu 2016 | GAPIT | 5.31E-05 | 6.1 |
|  |  |  |  | Xindu 2016 | TASSEL | 1.86E-04 | 6.4 |
|  | *IWB35570* | 558.1 | C/T | Wuhan 2017 | GAPIT | 9.86E-04 | 3.8 |
|  |  |  |  | Wuhan 2017 | TASSEL | 6.31E-04 | 5.0 |
|  | *IWA2218* | 566.6 | C/T | Xindu 2016 | GAPIT | 4.95E-04 | 4.5 |
|  |  |  |  | Wuhan 2017 | TASSEL | 7.21E-04 | 5.0 |
|  | *IWB58303* | 575.9 | A/C | Wuhan 2014 | GAPIT | 9.79E-04 | 4.4 |
|  |  |  |  | Wuhan 2017 | TASSEL | 7.56E-04 | 4.9 |
| 5B | *IWB60703* | 700.2 | A/G | Xindu 2016 | GAPIT | 7.17E-04 | 4.2 |
|  |  |  |  | Xindu 2016 | TASSEL | 5.36E-04 | 5.3 |
| 6B | *IWB58200* | 712.2 | A/C | Wuhan 2014 | GAPIT | 9.54E-04 | 4.4 |
|  |  |  |  | Wuhan 2014 | TASSEL | 1.37E-04 | 7.1 |
| 7B | *IWB9137* | 232.3 | A/G | Xindu 2016 | GAPIT | 3.03E-04 | 4.8 |
|  |  |  |  | Xindu 2016 | TASSEL | 2.88E-04 | 5.9 |
|  | *IWB24846* | 613.3 | A/C | Pixian 2016 | GAPIT | 5.04E-04 | 4.6 |
|  |  |  |  | Pixian 2016 | TASSEL | 5.39E-04 | 5.5 |
|  | *IWB71466* | 701.2 | A/G | Wuhan 2019 | GAPIT | 9.95E-04 | 3.8 |
|  |  |  |  | Wuhan 2019 | TASSEL | 7.66E-04 | 5.0 |
|  | *IWB38836* | 708.1 | C/T | Wuhan 2019 | GAPIT | 6.27E-05 | 5.7 |
|  |  |  |  | Wuhan 2019 | TASSEL | 2.90E-04 | 6.0 |
|  | *IWB45872* | 718.5 | C/T | Wuhan 2019 | GAPIT | 4.55E-04 | 4.3 |
|  |  |  |  | Wuhan 2019 | TASSEL | 2.70E-04 | 5.9 |
|  | *IWB7408* | 743.6 | G/T | Xindu 2016 | GAPIT | 4.80E-04 | 4.5 |
|  |  |  |  | Xindu 2016 | TASSEL | 3.25E-04 | 5.6 |
| 7D | *IWA7717* | 611.8 | C/T | Wuhan 2019 | GAPIT | 3.54E-04 | 4.5 |
|  |  |  |  | Wuhan 2019 | TASSEL | 4.66E-04 | 5.4 |

^a^ Favorable allele (SNP) is underlined; ^b^ Percentage of phenotypic variance explained by the QTL.
