# Supplementary material for: Genome-wide association analysis of stripe rust resistance in modern Chinese wheat
Source: BMC Plant Biol. 2020 Oct 27;20:491. doi: 10.1186/s12870-020-02693-w (PMC7590722; doi:10.1186/s12870-020-02693-w)

**Additional file 6** Quantile-quantile plot for stripe rust maximum disease severity in 240 wheat accessions analyzed using the mixed linear model with Tassel


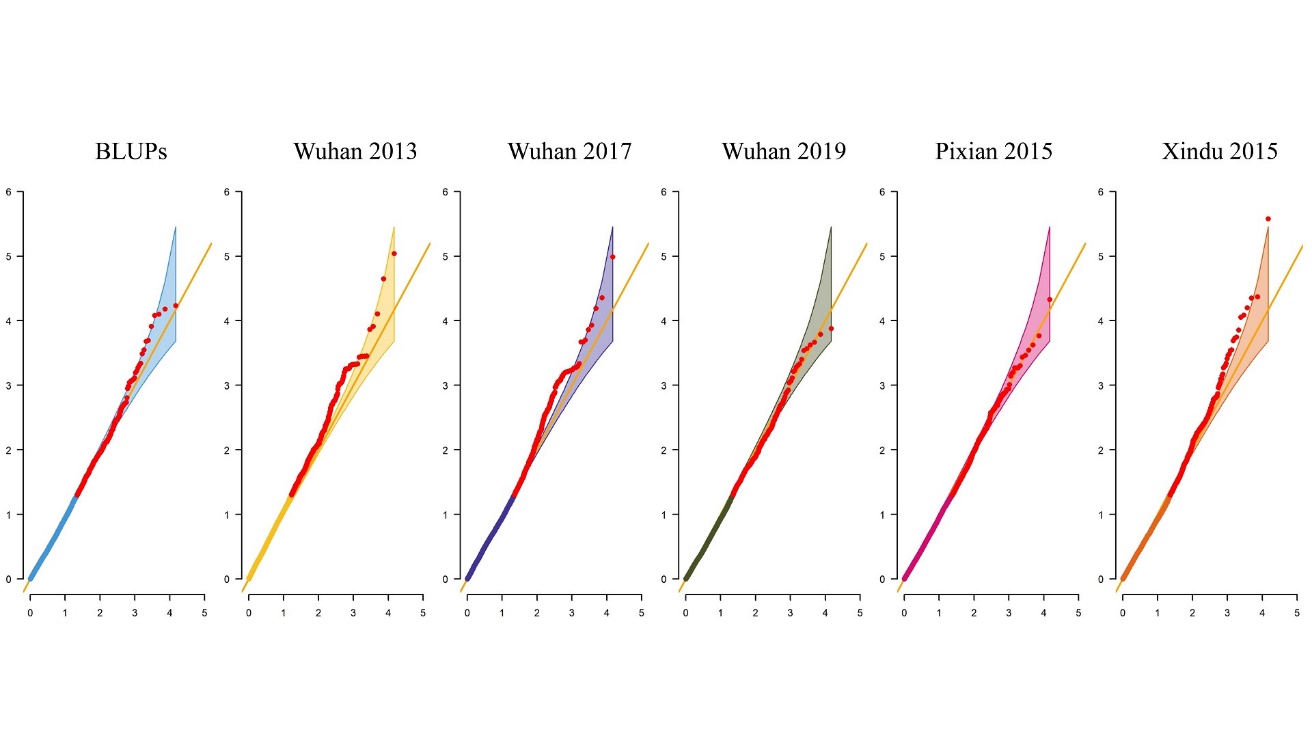

Supplement: Supplementary file 6 — Additional file 6. Quantile-quantile plot for stripe rust maximum disease severity in 240 wheat accessions analyzed using the mixed linear model with Tassel. [file 12870_2020_2693_MOESM6_ESM.doc]
