# Supplementary material for: Genome-wide association analysis of stripe rust resistance in modern Chinese wheat
Source: BMC Plant Biol. 2020 Oct 27;20:491. doi: 10.1186/s12870-020-02693-w (PMC7590722; doi:10.1186/s12870-020-02693-w)
Supplement: Supplementary file 8 — Additional file 8. Profiles of 11 penta-primer amplification refractory mutation system markers for mapped stripe rust resistance loci. [file 12870_2020_2693_MOESM8_ESM.doc]

**Additional file 8** Profiles of 11 penta-primer amplification refractory mutation system markers for mapped stripe rust resistance loci


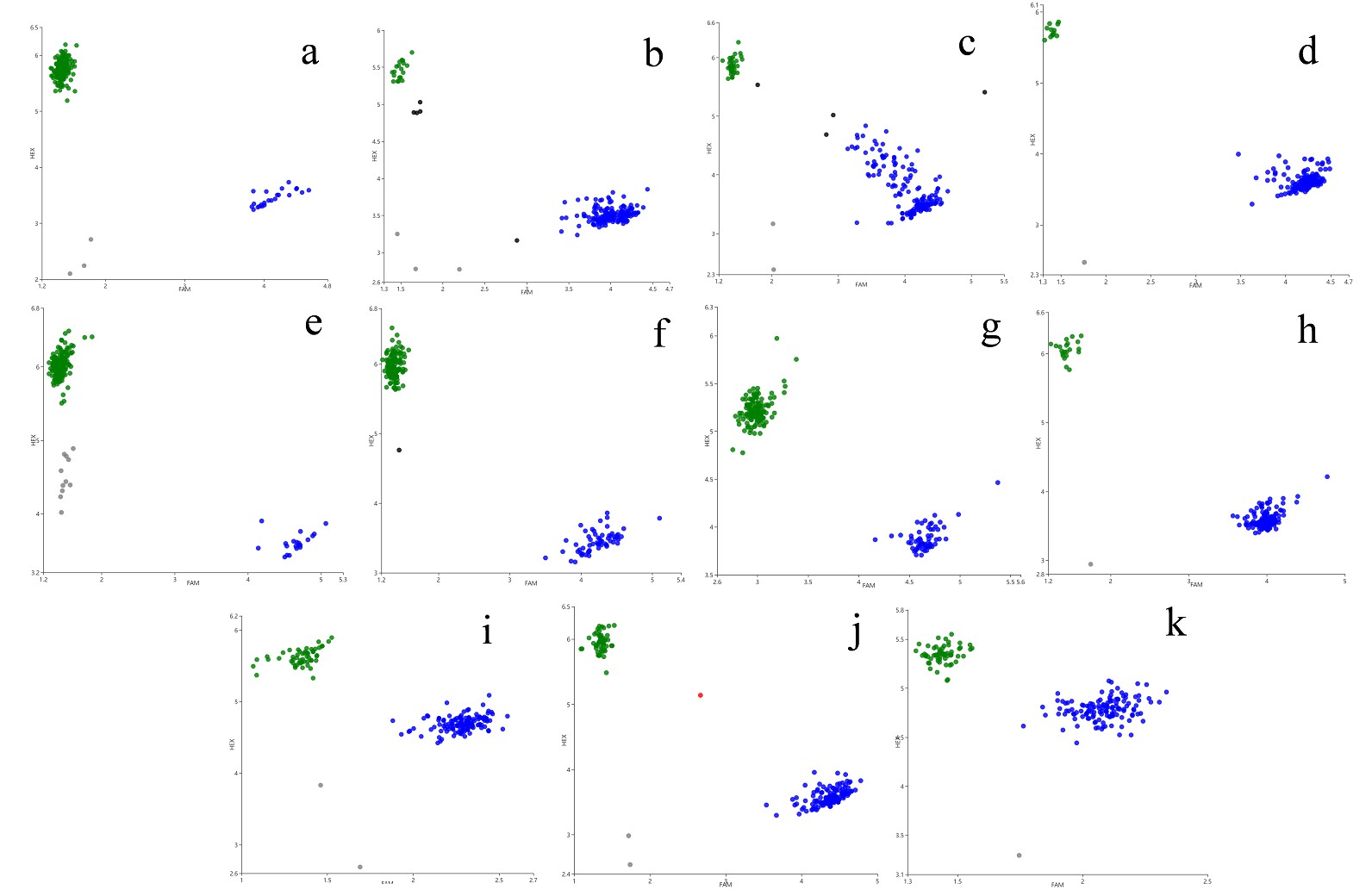


(a) *PARMS_IWA1787*, *QYr.hbaas-1DS*, green dots: resistant, blue dots: susceptible;

(b) *PARMS_IWA1788*, *QYr.hbaas-1DS*, green dots: susceptible, blue dots: resistant;

(c) *PARMS_IWB2650*, *QYr.hbaas-1DS*, green dots: susceptible, blue dots: resistant;

(d) *PARMS_IWA586*, *QYr.hbaas-2BL*, green dots: susceptible, blue dots: resistant;

(e) *PARMS_IWB12253*, *QYr.hbaas-3BS*, green dots: susceptible, blue dots: resistant;

(f) *PARMS_IWB73717*, *QYr.hbaas-4BL.1*, green dots: resistant, blue dots: susceptible.

(g) *PARMS_IWB27742*, *QYr.hbaas-4BL.1*, green dots: resistant, blue dots: susceptible;

(h) *PARMS_IWB63337*, *QYr.hbaas-4BL.2*, green dots: susceptible, blue dots: resistant;

(i) *PARMS_IWB57491*, *QYr.hbaas-4BL.2*, green dots: susceptible, blue dots: resistant;

(j) *PARMS_IWB59718*, *QYr.hbaas-4BL.3*, green dots: susceptible, blue dots: resistant;

(k) *PARMS_IWB60233*, *QYr.hbaas-6DS*, green dots: susceptible, blue dots: resistant.
