# Supplementary material for: Genome-wide association analysis of stripe rust resistance in modern Chinese wheat
Source: BMC Plant Biol. 2020 Oct 27;20:491. doi: 10.1186/s12870-020-02693-w (PMC7590722; doi:10.1186/s12870-020-02693-w)
Supplement: Supplementary file 9 — Additional file 9. Primers for 11 penta-primer amplification refractory mutation system markers for seven stripe rust resistance loci. [file 12870_2020_2693_MOESM9_ESM.doc]

**Additional file 9** Primers for 11 penta-primer amplification refractory mutation system markers for seven stripe rust resistance loci

| QTL | Marker | Primer | Sequence (5’-3’) ^a^ |
| --- | --- | --- | --- |
| *QYr.hbaas-1DS* | *PARMS_IWA1787* | A | GAAGGTGACCAAGTTCATGCTGAACAAAATGGAAACGGGTCA |
|  |  | B | GAAGGTCGGAGTCAACGGATTGAACAAAATGGAAACGGGTCG |
|  |  | Common | TGGTCGGTGTTTCGGTGAAT |
|  | *PARMS_IWB1788* | A | GAAGGTGACCAAGTTCATGCTAGCCATTTGTTAATCCAACACAT |
|  |  | B | GAAGGTCGGAGTCAACGGATTAGCCATTTGTTAATCCAACACAC |
|  |  | Common | ATCCGTTCTTGCCTGCTCTC |
|  | *PARMS_IWB2650* | A | GAAGGTGACCAAGTTCATGCTTCCTTGCACCATGACCATGA |
|  |  | B | GAAGGTCGGAGTCAACGGATTTCCTTGCACCATGACCATGC |
|  |  | Common | TGGAACTTCGACGAACTGCA |
| *QYr.hbaas-2BL* | *PARMS_IWA586* | A | GAAGGTGACCAAGTTCATGCTGCTATGATATCTGGTCGCTGAAA |
|  |  | B | GAAGGTCGGAGTCAACGGATTGCTATGATATCTGGTCGCTGAAG |
|  |  | Common | GGGACCAATATTTCCTATATGCAC |
| *QYr.hbaas-3BS* | *PARMS_IWB12253* | A | GAAGGTGACCAAGTTCATGCTACGCCATCAATGACCTCACA |
|  |  | B | GAAGGTCGGAGTCAACGGATTACGCCATCAATGACCTCACG |
|  |  | Common | TGGCCTACTAGCTGCGAAAG |
| *QYr.hbaas-4BL.1* | *PARMS_IWB73717* | A | GAAGGTGACCAAGTTCATGCTTTTGTCGGGTTTTATTGCCTTT |
|  |  | B | GAAGGTCGGAGTCAACGGATTTTTGTCGGGTTTTATTGCCTTC |
|  |  | Common | ATGGGACACAGGTGTTACCTA |
|  | *PARMS_IWB27742* | A | GAAGGTGACCAAGTTCATGCTTGAGCATTTCATCATCGACTGT |
|  |  | B | GAAGGTCGGAGTCAACGGATTTGAGCATTTCATCATCGACTGC |
|  |  | Common | ACAAGGTAGGATGGAGAAACCC |
| *QYr.hbaas-4BL.2* | *PARMS_IWB63337* | A | GAAGGTGACCAAGTTCATGCTTTGCCCGGAAGCATGATATACTTAA |
|  |  | B | GAAGGTCGGAGTCAACGGATTTTGCCCGGAAGCATGATATACTTAG |
|  |  | Common | TTCAGAATACGCATTTAAGCAGCCG |
|  | *PARMS_IWB57491* | A | GAAGGTGACCAAGTTCATGCTGGCTCACTCTGACTGGTCT |
|  |  | B | GAAGGTCGGAGTCAACGGATTGGCTCACTCTGACTGGTCC |
|  |  | Common | CGACGAAGAGGAGGAGGC |
| *QYr.hbaas-4BL.3* | *PARMS_IWB59718* | A | GAAGGTGACCAAGTTCATGCTGAGCACCAGACCATCATTCTT |
|  |  | B | GAAGGTCGGAGTCAACGGATTGAGCACCAGACCATCATTCTC |
|  |  | Common | AGGAGAGGCCACCCTTTCTA |
| *QYr.hbaas-6DS* | *PARMS_IWB60233* | A | GAAGGTGACCAAGTTCATGCTAAGGAAGATATATACAACCTCGTCA |
|  |  | B | GAAGGTCGGAGTCAACGGATTAAGGAAGATATATACAACCTCGTCG |
|  |  | Common | ACCTTCGGTGACTATTATGTACAAG |

^a^ Tails for competitive primers are underlined.
