# Supplementary material for: Genome-wide association analysis of stripe rust resistance in modern Chinese wheat
Source: BMC Plant Biol. 2020 Oct 27;20:491. doi: 10.1186/s12870-020-02693-w (PMC7590722; doi:10.1186/s12870-020-02693-w)
Supplement: Supplementary file 10 — Additional file 10. Protocols for 11 developed markers for seven stripe rust resistance loci. [file 12870_2020_2693_MOESM10_ESM.doc]

**Additional file 10** Protocols for 11 developed markers for seven stripe rust resistance loci

| QTL | Marker name | PCR system | PCR program | Detection | Result calling | |
| --- | --- | --- | --- | --- | --- | --- |
|  |  |  |  |  | Resistance | Susceptibility |
| *QYr.hbaas-1DS* | *PARMS_IWA1787* | 5 μL PARMS Master Mix, 0.7 μL primer mix (A:B:C = 3:3:8), 2.0 μL DNA template (30 ng/μL) and 2.3 μL ddH_2_O. | 3 min at 94°C; 10 touchdown cycles with a 0.8°C drop for annealing and elongation per cycle (94°C for 20 s, 65°C for 1min); 28 cycles (94°C 20 s, 57°C 1 min). | Fluorescence signals were detected using the multifunctional microplate reader PHERAstar^Plus^ and analyzed using KlusterCaller software. | green dot | blue dot |
|  | *PARMS_IWA1788* |  |  |  | blue dot | green dot |
|  | *PARMS_IWB2650* |  |  |  | blue dot | green dot |
| *QYr.hbaas-2BL* | *PARMS_IWA586* |  |  |  | blue dot | green dot |
| *QYr.hbaas-3BS* | *PARMS_IWB12253* |  |  |  | blue dot | green dot |
| *QYr.hbaas-4BL.1* | *PARMS_IWB73717* |  |  |  | green dot | blue dot |
|  | *PARMS_IWB27742* |  |  |  | green dot | blue dot |
| *QYr.hbaas-4BL.2* | *PARMS_IWB63337* |  |  |  | blue dot | green dot |
|  | *PARMS_IWB57491* |  |  |  | blue dot | green dot |
| *QYr.hbaas-4BL.3* | *PARMS_IWB59718* |  |  |  | blue dot | green dot |
| *QYr.hbaas-6DS* | *PARMS_IWB60233* |  |  |  | blue dot | green dot |
