# Supplementary material for: Genome-wide association analysis of stripe rust resistance in modern Chinese wheat
Source: BMC Plant Biol. 2020 Oct 27;20:491. doi: 10.1186/s12870-020-02693-w (PMC7590722; doi:10.1186/s12870-020-02693-w)
Supplement: Supplementary file 11 — Additional file 11. Candidate genes for stripe rust resistance QTLs. [file 12870_2020_2693_MOESM11_ESM.doc]

**Additional file 11** Candidate genes for stripe rust resistance QTLs

| QTL | Gene ID | Position (Mb) | Annotation ^a^ | Relative expression level ^b^ |
| --- | --- | --- | --- | --- |
| *QYr.hbaas-1BS* | TraesCS1B01G020600 | 9.6 | Receptor-like kinase | 1.8 (72 hpi) |
|  | TraesCS1B01G020900 | 9.7 | NBS-LRR disease resistance protein-like protein | 1.8 (72 hpi) |
|  | TraesCS1B01G022100 | 10.0 | NBS-LRR disease resistance protein-like protein | 1.2 (48 hpi) |
|  | TraesCS1B01G022200 | 10.1 | NBS-LRR disease resistance protein-like protein | 1.2 (48 hpi) |
|  | TraesCS1B01G022400 | 10.1 | Receptor-like kinase protein | 1.4 (72 hpi) |
| *QYr.hbaas-1DS* | TraesCS1D01G019600 | 8.6 | Disease resistance protein RPM1 | 0.8 (48 hpi) |
|  | TraesCS1D01G019700 | 8.6 | Disease resistance protein RPM1 | 1.1 (72 hpi) |
|  | TraesCS1D01G018700 | 8.2 | NBS-LRR disease resistance protein | 1.3 (24 hpi) |
| *QYr.hbaas-2AS* | TraesCS2A01G029800 | 13.4 | NBS-LRR-like resistance protein | 0.7 (24 hpi) |
|  | TraesCS2A01G030000 | 13.8 | NBS-LRR-like resistance protein | 1.0 (48 hpi) |
|  | TraesCS2A01G030200 | 13.8 | NBS-LRR-like resistance protein | 0.8 (24 hpi) |
|  | TraesCS2A01G031000 | 14.3 | NBS-LRR-like resistance protein | 1.0 (72 hpi) |
| *QYr.hbaas-2BL* | TraesCS2B01G317500 | 453.0 | Disease resistance protein (TIR-NBS-LRR class) family | 1.2 (72 hpi) |
| *QYr.hbaas-3AL* | TraesCS3A01G274000 | 502.8 | Protein kinase | 1.3 (24 hpi) |
| *QYr.hbaas-3BS* | TraesCS3B01G021400 | 9.1 | ATP-binding cassette sub-family A member 1 | 0.7 (24 hpi) |
|  | TraesCS3B01G021100 | 8.8 | Glycosyltransferase | 2.1 (48 hpi) |
|  | TraesCS3B01G021700 | 9.2 | Glycosyltransferase | 1.3 (24 hpi) |
|  | TraesCS3B01G021900 | 9.4 | Glycosyltransferase | 1.0 (72 hpi) |
|  | TraesCS3B01G022000 | 9.4 | Glycosyltransferase | 1.5 (48 hpi) |
| *QYr.hbaas-4BL.3* | TraesCS4B01G294300 | 579.7 | Kinase-like protein | 1.4 (48 hpi) |
| *QYr.hbaas-4DL* | TraesCS4D01G312300 | 478.2 | Protein kinase | 1.0 (72 hpi) |
|  | TraesCS4D01G310900 | 477.9 | receptor kinase 1 | 1.1 (24 hpi) |
|  | TraesCS4D01G312400 | 478.3 | Receptor-like kinase | 1.1 (72 hpi) |
| *QYr.hbaas-6DS* | TraesCS6D01G015500 | 6.4 | Leucine-rich repeat receptor-like protein kinase family protein | 1.3 (48 hpi) |
|  | TraesCS6D01G015600 | 6.4 | Leucine-rich repeat receptor-like protein kinase family protein | 1.0 (48 hpi) |
|  | TraesCS6D01G013600 | 5.6 | Receptor-like protein kinase | 1.7 (72 hpi) |
|  | TraesCS6D01G014300 | 6.0 | Stress response NST1-like protein | 6.0 (48 hpi) |

^a^ Gene annotations were referred to IWGSC RefSeq annotation v1.0 (www.wheatgenome.org);

^b^ The highest expressions of corresponding genes post-inoculation were compared to those before inoculation (0 hour post-inoculation, hpi). The time when the genes expressed at the highest level was shown in the brackets after the relative expression level. Gene expressions were referred to the public wheat expression database Triticeae Multi-omics Center (http://202.194.139.32) using the study performed by Zhang et al. (2014).
