# Supplementary material for: Genome-wide association analysis of stripe rust resistance in modern Chinese wheat
Source: BMC Plant Biol. 2020 Oct 27;20:491. doi: 10.1186/s12870-020-02693-w (PMC7590722; doi:10.1186/s12870-020-02693-w)
Supplement: Supplementary file 12 — Additional file 12. Reported QTLs/genes, linked markers and their physical positions used to align with QTLs mapped in the present study. [file 12870_2020_2693_MOESM12_ESM.doc]

**Additional file 12** Reported QTLs/genes, linked markers and their physical positions used to align with QTLs mapped in the present study

| Chromosome arm | Reported QTLs/genes | Linked markers | Physical Position (Mb)^a^ | Reference |
| --- | --- | --- | --- | --- |
| 1BS | *YrH62* | *IWB11553 - IWB9149* | 280.2 - 329.4 | [64] |
|  | *QYr.sicau-1B* | *1254926* | 145.7 | [18] |
|  | *QLr.ifa-1B* | *wPt-3103 - Xgwm11* | 51.3 - 216 | [65] |
|  | *QYrco.wpg-1B.2* | *Xbarc119* | 53.3 | [66] |
|  | *yrGn22* | *Xgwm18* | 222.6 | [67] |
|  |  | *IWA1191* | 57.7 | [68] |
|  | *Yr64* | *Xgwm413* | 73.1 | [21] |
|  | *Yr15* | *Xbarc8 - Xgwm273* | 26.7-212.5 |  |
|  | *YrMY41* | *Xwmc406 - Xgwm18* | 38.6 - 222.6 | [69] |
|  |  | *wsnp_BE405749B_Ta_2_1* | 9.1 | [22] |
|  | *QYr.nwafu-1BS.1* | *AX-109418656* | 17.5 | [23] |
|  | *QYr.nwafu-1BS.2* | *AX-108898523* | 39.8 |  |
|  | *QYr.nwafu-1BS.3* | *AX-110711112* | 203.1 |  |
| 1DS | *QYr.sicau-1D* | *Xgwm337* | 28.7 | [70] |
|  |  | *IWA980* | 36.3 | [45] |
|  | *QYr.nwafu-1DS.1* | *AX-94849392* | 2.5 | [23] |
|  | *QYr.nwafu-1DS.2* | *AX-108917513* | 134.5 |  |
|  | *QYrst.orr-1DS* | *Xbcd1434* | 16.8 | [24] |
|  | *QYr.caas-1DS* | *XUgwm353–Xgdm33b* | unknown | [49] |
|  | *QYr.sun-1D* | *Xwmc147* | 0.3 | [25] |
| 2AS | *Traes_2AS_6BC67DD45* | *S4_5007061* | 8.9 | [16] |
|  | *Traes_2AS_A477CDA77* | *S4_5287800* | 8.9 |  |
|  | *Traes_2AS_6CE6AB560* | *S4_7117805* | 8.9 |  |
|  |  | *IWB57199* | 12.7 | [15] |
|  | *Yr2A.1PBL* | *1206128|F|0-54:G>A-54:G>A* | 14.2 | [26] |
|  | *Yr2A.2PBL* | *3951942|F|0-8:A>G-8:A>G* | 16 |  |
|  | *Yr2A.3PBL* | *100247987|F|0-40:G>A-40:G>A* | 13.3 |  |
|  | *Yr2A.4PBL* | *2293684|F|0-18:C>T-18:C>T* | 14.4 |  |
|  | *QYr.tam-2A* | *wPt-3896 - wPt-6431* | 7.1-13.1 | [27] |
|  | *QYr.tsw-2A.3* | *IWB11136* | 18.6 | [28] |
|  | *QYr.tsw-2A.5* | *IWB65219* | 0.4 |  |
|  | *YrCH86* | *Xbarc124 - Xgwm636* | 5.7-6.7 | [29] |
|  |  | *IWA422* | 12.6 | [45] |
|  | *QYr.sun-2A* | *wPt-1041* | 8.1 | [31] |
|  | *QYr.ufs-2A* | *Xgwm636* | 6.7 | [32] |
|  | *YrR61* | *Xbarc124 - Xgwm359* | 3.8-9.2 | [33] |
|  |  | *1180242D* | 54.6 | [17] |
|  | *QYr.sicau-2A.3* | *Xbarc212* | 1.6 | [46] |
|  | *QYrPI182103.wgp-2AS* | *IWA5585 - Xgwm312* | 115.4-709 | [37] |
|  | *Yrzhong16-1* | *Xbarc212* | 1.6 | [71] |
|  | *QYr.tamu-2A1* | *IWA2526* | 21 | [72] |
|  | *QYr.nwafu-2AS* | *AX-108928357* | 1.5 | [23] |
|  | *QYrst.orr-2AS* | *wPt - 1657* | 47.2 | [24] |
| 2BL | *QYr.sicau-2B.1* | *1265469* | 732.1 | [46] |
|  | *QYr.sicau-2B.2* | *1006874* | 799.5 |  |
|  | *YrSP* | *Xwmc332* | 739.4 | [34] |
|  |  | *wsnp_JD_c744_1111659* | 580 | [39] |
|  | *QYr.sicau-2B* | *2278639* | 759 | [70] |
|  |  | *IWA1040* | 726 | [68] |
|  | *QYr.caas-2BL.2* | *Xcfd73-Xgwm47* | 685.8 | [73] |
|  | *QYr.spa-2B.1* | *Xwmc25* | 280.9 | [74] |
|  | *QYrqn.nwafu-2BL* | *AX-94507002* | 674 | [75] |
|  | *QYr.nwafu-2BL* | *AX-94915683* | 796.8 | [23] |
|  | *Yr7* | *Xwmc339* | 660 | [35] |
|  | *QYraq.cau-2BL* | *Xwmc332* | 671 | [76] |
|  | *QYr.inra-2BL* | *Xbarc101 - Xgwm120* | 615.8-621 | [77] |
|  | *QYr.caas-2BL* | *XwPt-8460–XwPt-3755* | 693.7-733.2 | [49] |
| 3AL | *QYr.dms-3A* | *3A_d1103494* | 647 | [36] |
|  | *QYrPI182103.wgp-3AL* | *Xgwm2 - IWA899* | 60.2-490.3 | [37] |
|  | *QYrdr.wgp-3AL* | *IWA6834* | 438.3 | [38] |
|  |  | *wsnp_JD_c14691_14352459* | 533 | [39] |
|  |  | *IWB11855* | 692.4 | [40] |
|  |  | *wsnp_RFL_Contig4814_5829093* | 394.8 | [22] |
|  | *QYr.nwafu-3AL* | *AX-109327388* | 721.3 | [23] |
|  | *QYrst.orr-3AL* | *wPt - 1652* | unknown | [24] |
|  | *QYr.sicau-3A* | *1131042* | unknown | [18] |
| 3BS | *Qyrlov.nwafu-3BS* | *IWB6491* | 14 | [78] |
|  |  | *IWA5202* | 3.9 | [45] |
|  | *QYr.ifa-3BS* | *Xbarc133* | 7.6 | [65] |
|  | *QYr.sicau-3B.1* | *Xgwm389* | 0.8 | [46] |
|  | *QYr.nwafu-3BS* | *AX-94545746* | 10.7 | [47] |
|  | *QYrsk.wgp-3BS* | *IWB67768* | 8.8 | [79] |
|  | *Yr57* | *gwm389* | 0.8 | [80] |
|  | *QYr.spa-3B.1* | *Xbarc147* | 7.2 | [74] |
|  | *QYr.spa-3B.2* | *wPt-666738* | 35.6 |  |
|  | *QYr.ar.3BS* | *Xbarc147* | 7.2 | [81] |
|  | *Qyrlov.nwafu-3BS* | *IWB57990 - IWB6491* | 14 | [78] |
|  | *QYrhm.nwafu-3BS* | *Xbarc87 - IWB39254* | 14.4 | [42] |
|  | *QYr.nafu-3BS* | *Xgwm533.2 - Xgwm493* | 35.3-6.5 | [82] |
|  | *QYrto.swust-3BS* | *AX-94509749-AX-94998050* | 34.3-6.4 | [83] |
|  | *QYr.nwafu-3BS* | *AX-110739849* | 8.8 | [23] |
|  | *QYr.uga-3BS.1* | *wPt-2557 - Xbarc133* | 7.6 | [33] |
|  | *QYr.uga-3BS.3* | *wPt-1612–wPt-7486* | 60.4 |  |
|  | *Sr2/Yr30* | *bex2f - MSF_2f* | 6.1-6.6 | [41] |
|  | *QYr.sun-3B* | *wPt-9577* | unknown | [31] |
|  | *QYrbr.wpg-3B.2* | *wPt-9432* | 187.5 | [66] |
|  |  | *wsnp_Ex_c1558_2976128* | 20 | [39] |
|  | *Yrwh2* | *Xwmc540 - Xgwm566* | 133-77.7 | [84] |
|  | *Yrns-B1* | *Xgwm493 - Xgwm533* | 31.7-35.3 | [85] |
|  | *QYr.sun-3B* | *wPt-6802* | 43.7 | [86] |
|  | *QYr.inra-3BS* | *Xgwm533* | 35.3 | [87] |
| 4BL | *Qyr.wpg-4B.1* | *IWA4348* | 531.1 | [88] |
|  | *Yr62* | *Xgwm192-Xgwm251* | 509.0-568.6 | [43] |
|  | *QYrhm.nwafu-4B* | *AX-111150955-Xgwm251* | 523.4-568.6 | [42] |
|  | *QYr.caas-4BL* | *Xgwm165–Xgwm149* | *509.0-544.6* | [89] |
|  | *QYr.ar-4BL* | *IWA4640* | unknown | [81] |
|  | *QYr.nwafu-4BL* | *AX-110963704—AX-110564812* | 189.7-192 | [90] |
|  | *QYr.nwafu-4BL* | *AX-89362089* | 640.1 | [23] |
|  | *QPst.jic-4B* | *Xcfd039* | 610.6 | [91] |
|  | *QPst.jic-4B* | *Xwmc652 - Xwmc692* | 592.6-622.3 | [92] |
|  |  | *Xgwm495* | 482.8 | [93] |
|  | *QYr.sun-4B* | *wPt-8543- Xwmc238* | 250 | [25] |
| 4DL | *QYr.caas-4DL.2* | *Xbarc98-Xbarc1148* | 256.7-325 | [73] |
|  |  | *Xwmc399* | 484.7 | [44] |
|  | *QYr.caas-4DL* | *Xwmc331-Xgwm165* | 453.3 | [94] |
| 6DS | *QYr.sicau-6D* | *Xcfd188* | 238.1 | [46] |
|  | *QYr.ucw-6D* | *IWA167* | 105.5 | [45] |
|  | *QYr.ufs-6D* | *Xgwm325–Xbarc175* | 79.9-411.9 | [32] |
| 7BL | *Qyrsicau-7BL* | *AX-110518451* | 711.4 | [19] |
|  | *QYr.nwafu-7BL* | *AX-110028937* | 707.7 | [47] |
|  | *Qyr.saas-7B* | *Marker66294 - Marker66313* | 678.6-706.8 | [48] |
|  | *QYr.nwafu-7BL* | *AX-109913626* | 709 | [23] |
|  | *QYr.caas-7BL.1* | *Xbarc176 - wPt8106* | 557 | [49] |
|  | *QYr.caas-7BL.2* | *Xgwm577 - wPt-4300* | 711.2 |  |
|  |  | *wPt-4342-wPt-8921* | 693.3 | [50] |
|  |  | *IWB58601* | 732.7 | [15] |
|  |  | *1138514D* | 721.1 | [17] |
|  | *QYrel.wgp-7BL* | *IWB4739* | 483.8 | [95] |
|  |  | *IWA3415* | 732.7 | [68] |
|  | *yrMY37* | *Xgwm297- Xbarc267* | 237.5-377.1 | [96] |
|  | *QyrPI182103.wgp-7BL/Yr79* | *Xbarc72 - Xwmc335* | 214.1-233.2 | [37] |
|  | *Yr52* | *Xcfa2040-Xbarc182* | 718.4-732.4 | [97] |
|  |  | *XP32/M59-Xgwm344* | 727.4 | [98] |
|  |  | *Xwmc166* | 719.8 | [44] |
|  | *YrC591* | *Xmag1714 - Xbarc182* | 732.4 | [99] |
|  | *Yr59* | *Xwgp5175 - Xbarc32* | 723.9-732.4 | [100] |

^a^ IWGSC RefSeq v1.0; https://wheat.pw.usda.gov.

**References**

64. Wu J, Wang Q, Xu L, Chen X, Li B, Mu J, Zeng Q, Huang L, Han D, Kang Z. Combining SNP genotyping array with bulked segregant analysis to map a gene controlling adult-plant resistance to stripe rust in wheat line 03031-1-5 H62. Phytopathology. 2017;108(1):103–13.

65. Buerstmayr M, Matiasch L, Mascher F, Vida G, Ittu M, Robert O, Holdgate S, Flath K, Neumayer A, Buerstmayr H. Mapping of quantitative adult plant field resistance to leaf rust and stripe rust in two European winter wheat populations reveals co‑location of three QTL conferring resistance to both rust pathogens. Theor Appl Genet. 2014;127(9):2011–2028.

66.Case AJ, Naruoka Y, Chen XM, Garland-Campbell KA, Zemetra RS, Carte AH. Mapping stripe rust resistance in a Brundage×Coda winter wheat recombinant inbred line population. PLoS One. 2014;9(3): e91758.

67. Li Q, Ma DF, Li Q, Fan Y, Shen XX, Jing JX, Wang BT, Kang ZS. Genetic Analysis and Molecular Mapping of a Stripe Rust Resistance Gene in Chinese Wheat Differential Guinong 22. J Phytopathology. 2016;164(7-8):476-84.

68. Muleta KT, Bulli P, Rynearson S, Chen X, Pumphrey M. Loci associated with resistance to stripe rust (*Puccinia striiformis* f. sp. *tritici*) in a core collection of spring wheat (*Triticum aestivum*). PLoS One. 2017;12(6): e0179087.

69.Ren Y, Li S, Wei Y, Zhou Q, Du X, He Y, Zheng Y. Molecular mapping of a stripe rust resistance gene in Chinese wheat cultivar Mianmai 41. J Integr Agr. 2015;14(2):295–304.

70. Long L, Yao F, Yu C, Ye X, Cheng Y, Wang Y, Wu Y, Li J, Wang J, Jiang Q, Li W, Ma J, Liu Y, Deng M, Wei Y, Zheng Y and Chen G. Genome-wide association study for adult-plant resistance to stripe rust in Chinese wheat landraces (*Triticum aestivum* L.) from the Yellow and Huai River Valleys. Front Plant Sci. 2019;10:596.

71. Ma D, Li Q, Zhao M. Inheritance and molecular mapping of stripe rust resistance genes in Chinese winter wheat Zhongliang 16. Crop Prot. 2015;74:51–5.

72. Yang Y, Basnet BR, Ibrahim AMH, Rudd J, Chen X, Bowden RL, Xue Q, Wang S, Johnson CD, Metz R, Mason RE, Hays DB, Liu S. Developing KASP markers on a major stripe rust resistance QTL in a popular wheat TAM 111 using 90K array and genotyping-by-sequencing SNPs. Crop Sci. 2018;59(1), https://doi.org/10.215/cropsci2018.05.0394.

73. Ren Y, Liu L, He Z, Wu L, Bai B, Xia X. QTL mapping of adult-plant resistance to stripe rust in a ‘Lumai 21 × Jingshuang 16’ wheat population. Plant Breeding. 2015;134(5), https://doi.org/10.1111/pbr.12290.

74. Singh A, Knox RE, DePauw RM, Singh AK, Cuthbert RD, Campbell HL, Shorter S, Bhavan S. Stripe rust and leaf rust resistance QTL mapping, epistatic interactions, and co‑localization with stem rust resistance loci in spring wheat evaluated over three continents. Theor Appl Genet. 2014;127(11):2465–77.

75. Zeng Q, Wu J, Liu S, Chen X, Yuan F, Su P, Wang Q, Huang S, Mu J, Han D, Kang Z. Genome-wide mapping for stripe rust resistance loci in common wheat cultivar Qinnong 142. Plant Dis. 2018;103:439–47.

76. Guo Q, Zhang Z, Xu Y, Li G, Feng J, Zhou Y. Quantitative trait loci for high-temperature adult-plant and slow-rusting resistance to *Puccinia striiformis* f. sp. *tritici* in wheat cultivars. Phytopathology. 2008;98(7):803–9.

77. Mallard S, Gaudet D, Aldeia A, Abelard C, Besnard AL, Sourdille P, Dedryver F. Genetic analysis of durable resistance to yellow rust in bread wheat. Theor Appl Genet. 2005;110(8):1401–9.

78. Wu J, Wang Q, Chen X, Liu S, Li H. Development and validation of KASP-SNP markers for QTL underlying resistance to stripe rust in common wheat cultivar P10057. Plant Dis. 2017;101(12):2079–87.

79. Liu L, Yuan C, Wang M, See DR, Zemetra RS, Chen X. QTL analysis of durable stripe rust resistance in the North American winter wheat cultivar Skiles. Theor Appl Genet. 2019;132(6):1677–91.

80. Randhawa MS, Bariana HS, Mago R, Bansal UK. Mapping of a new stripe rust resistance locus *Yr57* on chromosome 3BS of wheat. Mol Breeding. 2015;35(2):65.

81. Subramanian NK, Mason RE, Milus EA, Moon DE. Characterization of two adult-plant stripe rust resistance genes on chromosomes 3BS and 4BL in soft red winter wheat. Crop Sci. 2015;56(1), https://doi.org/10.2135/cropsci2015.01.0043

82. Zhou X, Zhang Y, Zeng Q, Chen X, Han D, Huang L, Kang Z. Identification of QTL for adult plant resistance to stripe rust in Chinese wheat landrace Caoxuan 5. Euphytica. 2015;204(3):627–34.

83. Zhou X, Hu T, Li X, Yu M, Li Y, Yang S, Huang K, Han D, Kang Z. Genome‑wide mapping of adult plant stripe rust resistance in wheat cultivar Toni. Theor Appl Genet. 2019;132:1693–704.

84. Zhou X, Han D, Gou H, Wang Q, Zeng Q, Yuan F, Zhan G, Huang L, Kang Z. Molecular mapping of a stripe rust resistance gene in wheat cultivar Wuhan 2. Euphytica. 2014;196:251–9.

85. Börner A, Röder MS, Unger O, Meinel A. The detection and molecular mapping of a major gene for non-specific adult-plant disease resistance against stripe rust (*Puccinia striiformis*) in wheat. Theor Appl Genet. 2000;100(7):1095–9.

86. Bariana HS, Bansal UK, Schmidt A, Lehmensiek A, Kaur J, Miah H, Howes N, McIntyre CL. Molecular mapping of adult plant stripe rust resistance in wheat and identification of pyramided QTL genotypes. Euphytica. 2010;176(2):251–60.

87. Dedryver F, Paillard S, Mallard S, Robert O, Trottet M, Nègre S, Verplancke G, Jahi J. Characterization of genetic components involved in durable resistance to stripe rust in the bread wheat 'Renan'. Phytopathology. 2009;99(8):968–73.

88. Naruoka Y, Garland-Campbell KA, Carter AH. Genome‑wide association mapping for stripe rust (*Puccinia striiformis* F. sp. *tritici*) in US Pacific Northwest winter wheat (*Triticum aestivum* L.). Theor Appl Genet. 2015;128(6):1083–101.

89. Lu Y, Lan C, Liang S, Zhou X, Liu D, Zhou G, Lu Q, Jing J, Wang M, Xia X, He Z. QTL mapping for adult-plant resistance to stripe rust in Italian common wheat cultivars Libellula and Strampelli. Theor Appl Genet. 2009;119(8):1349-59.

90. Wu J, Huang S, Zeng Q, Liu S, Wang Q, Mu J, Yu S, Han D, Kang Z. Comparative genome‑wide mapping versus extreme pool‑genotyping and development of diagnostic SNP markers linked to QTL for adult plant resistance to stripe rust in common wheat. Theor Appl Genet. 2018;131:1777–92.

91. Jagger LJ, Newell C, Berry ST, MacCormack R, Boyd LA. The genetic characterisation of stripe rust resistance in the German wheat cultivar Alcedo. Theor Appl Genet. 2011;122(4):723–33.

92. Melichar JPE, Berry S, Newell C, MacCormack R, Boyd LA. QTL identification and microphenotype characterization of the developmentally regulated yellow rust resistance in the UK wheat cultivar Guardian. Theor Appl Genet. 2008;117:391–9.

93. William HM, Singh RP, Huerta-Espino J, Palacios G, Suenaga K. Characterization of genetic loci conferring adult plant resistance to leaf rust and stripe rust in spring wheat. Genome. 2006;49(8):977–90.

94. Ren Y, Li Z, He Z, Wu L, Bai B, Lan C, Wang C, Zhou G, Zhu H, Xia X. QTL mapping of adult-plant resistances to stripe rust and leaf rust in Chinese wheat cultivar Bainong 64. Theor Appl Genet. 2012;125(6):1253–62.

95. Liu L, Wang M, Feng J, See DR, Chen X. Whole genome mapping of stripe rust resistance QTL and race-specificity related to resistance reduction in winter wheat cultivar Eltan. Phytopathology. 2019;109(7):1226–35.

96. Ren Y, Li S, Xia X, Zhou Q, He Y, Wei Y, Zheng Y, He Z. Molecular mapping of a recessive stripe rust resistance gene *yrMY37* in Chinese wheat cultivar Mianmai 37. Mol Breeding. 2015;35:97.

97. Ren R, Wang M, Chen X, Zhang Z. Characterization and molecular mapping of *Yr52* for high-temperature adult-plant resistance to stripe rust in spring wheat germplasm PI 183527. Theor Appl Genet. 2012;125(5):847–57.

98. Rosewarne GM, Singh RP, Espino JH, Rebetzke GJ. Quantitative trait loci for slow-rusting resistance in wheat to leaf rust and stripe rust identified with multi-environment analysis. Theor Appl Genet. 2008;116(7):1027–34.

99. Xu H, Zhang J, Zhang P, Qie Y, Niu Y, Li H, Ma P, Xu Y, An D. Development and validation of molecular markers closely linked to the wheat stripe rust resistance gene *YrC591* for marker-assisted selection. Euphytica. 2014;198(3):317-23.

100. Zhou X, Wang M, Chen X, Lu Y, Kang Z, Jing J. Identification of *Yr59* conferring high‑temperature adult‑plant resistance to stripe rust in wheat germplasm PI 178759. Theor Appl Genet. 2013;127(4):935–45.
